# A Modular Core–Shell Nanoparticle Platform for Dual‐Modal MRI–Luminescence With High Relaxivity

**DOI:** 10.1002/smll.74389

**Published:** 2026-07-03

**Authors:** Catherine J. Ashton, Jonathan Martinelli, Sara Camorali, Thomas R. Berki, Amy J. Managh, Constantina Sofroniou, Lorenzo Tei, Helen Willcock, Stephen J. Butler

**Affiliations:** ^1^ Department of Chemistry Loughborough University Loughborough UK; ^2^ Department of Materials Loughborough University Loughborough UK; ^3^ Department of Science and Technological Innovation Università del Piemonte Orientale Alessandria Italy

**Keywords:** imaging agent, luminescence, macromolecule, MRI contrast agent, nanoparticle

## Abstract

Bimodal imaging agents that combine MRI and optical signals can improve imaging and diagnostic precision by providing complementary anatomical and molecular information. Macromolecular MRI agents can deliver higher relaxivities than small‐molecule analogues by using controlled architectures that slow rotational motion and enhance Gd(III)–water interactions. The combination of bimodal functionality with macromolecular designs represents a highly promising route to new imaging agents, but its potential has not yet been realized due to safety concerns over possible unchelated metals and difficulties in achieving reproducible large‐scale production. Here, we present a simple, reproducible method to directly incorporate isostructural Gd(III) and Tb(III) complexes into amphiphilic core–shell block copolymer nanoparticles using a single macrocyclic methacrylate ligand. This modular design enables controlled spatial positioning of each MRI‐ and luminescence agent, achieving high relaxivity up to 30.2 mM^−1^s^−1^ per Gd(III) and long‐lived (>1.30 ms) luminescence, providing a tunable platform for dual‐modal MRI–luminescence applications.

## Graphical abstract

A modular core–shell nanoparticle platform is presented that combines high‐relaxivity magnetic resonance imaging (MRI) and long‐lived luminescence within a single, well‐defined architecture. Spatially controlled gadolinium(III) and terbium(III) complexes deliver reproducible dual‐modal agents with benchmark MRI performance.

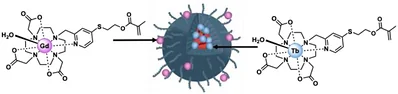

## 1 Introduction

Magnetic Resonance Imaging (MRI) offers non‐invasive 2D and 3D anatomical imaging with sub‐millimeter spatial resolution, unlimited penetration depth, and excellent soft tissue contrast. These features make MRI invaluable for neurological, cardiovascular, and oncological diagnostics. However, MRI suffers low intrinsic sensitivity, which can be enhanced using contrast agents (CAs) to increase the relaxation rate of local water protons [1]. Most clinically approved CAs are based on low molecular weight macrocyclic gadolinium(III) complexes [2, 3, 4], such as Gd‐DOTA which has a relaxivity of *r*_1_ = 4.7 mM^−1^s^−1^ (20 MHz, 298 K), far from the theoretical maximum of 100 mM^−1^s^−1^ (for mono‐aqua complexes) [5, 6].

Dual imaging agents, which combine techniques such as MRI and optical imaging, ensure identical biodistribution for each component and integrate the strengths of different modalities, enhancing diagnostic capabilities [7, 8, 9, 10]. MRI's high resolution complements the high sensitivity of optical imaging probes, allowing visualization of the same structures at different resolutions and depths. Such dual agents can guide surgeons with optical imaging and confirming tumor removal post‐surgery with MRI [9]. Several small molecule dual agents have been developed, combining a Gd(III) MRI‐active moiety with an organic or inorganic emissive component for dual imaging [11, 12, 13]. Lanthanide(III) complexes, especially those of Eu(III) and Tb(III), represent an attractive class of luminescence imaging agents, due to their sharp visible emission bands and long‐lived excited states [14, 15, 16, 17]. However, the high hydrophobicity of small molecule dual‐imaging agents can cause them to accumulate in fatty tissue, reducing their interaction with water molecules and limiting the MRI signal [18, 19].

Macromolecular systems present an alternative strategy to enhance relaxivity and combine the benefits of macromolecular and bimodal imaging systems [20, 21, 22, 23, 24, 25], although their in vivo behavior and biodistribution are influenced by particle size, surface chemistry and architecture [26]. Macromolecules offer enhanced sensitivity in MRI by slowing molecular tumbling [27, 28], while hydrophilic shell architectures can reduce non‐specific protein and membrane interactions that commonly limit the bioavailability of small‐molecule hydrophobic agents. Furthermore, they can provide complementary imaging modalities into a single system. Importantly, the amount of MRI‐active and optically active components can be controlled to optimize signal and sensitivity. Strategies include conjugating Gd(III) chelates and organic/inorganic fluorophores to linear and branched polymers, dendrimers, micelles, liposomes, peptide‐based coiled coils, or non‐covalent association with biomolecules or nano‐assembled capsules [5, 29, 30, 31, 32, 33, 34, 35, 36, 37]. Some macromolecular CAs achieve relaxivities up to 40 mM^−1^s^−1^ [5], attributed predominantly to the slower tumbling and presence of multiple Gd(III) complexes within a single macromolecule.

Despite the advantages, challenges remain in achieving reproducible macromolecular CAs with controlled size, shape, and constitution. A second challenge lies in creating systems where the motion of the macromolecule is effectively coupled to the motion of multiple paramagnetic Gd(III) centers. One successful approach positions a single Gd(III) complex at the barycenter of a macromolecule, where the rotational correlation time is dictated by the macromolecule's motion [21]. Incorporating multiple Gd(III) centers can further enhance relaxivity, provided their local motion is coupled to the global motion of the particle.

**FIGURE 1:**
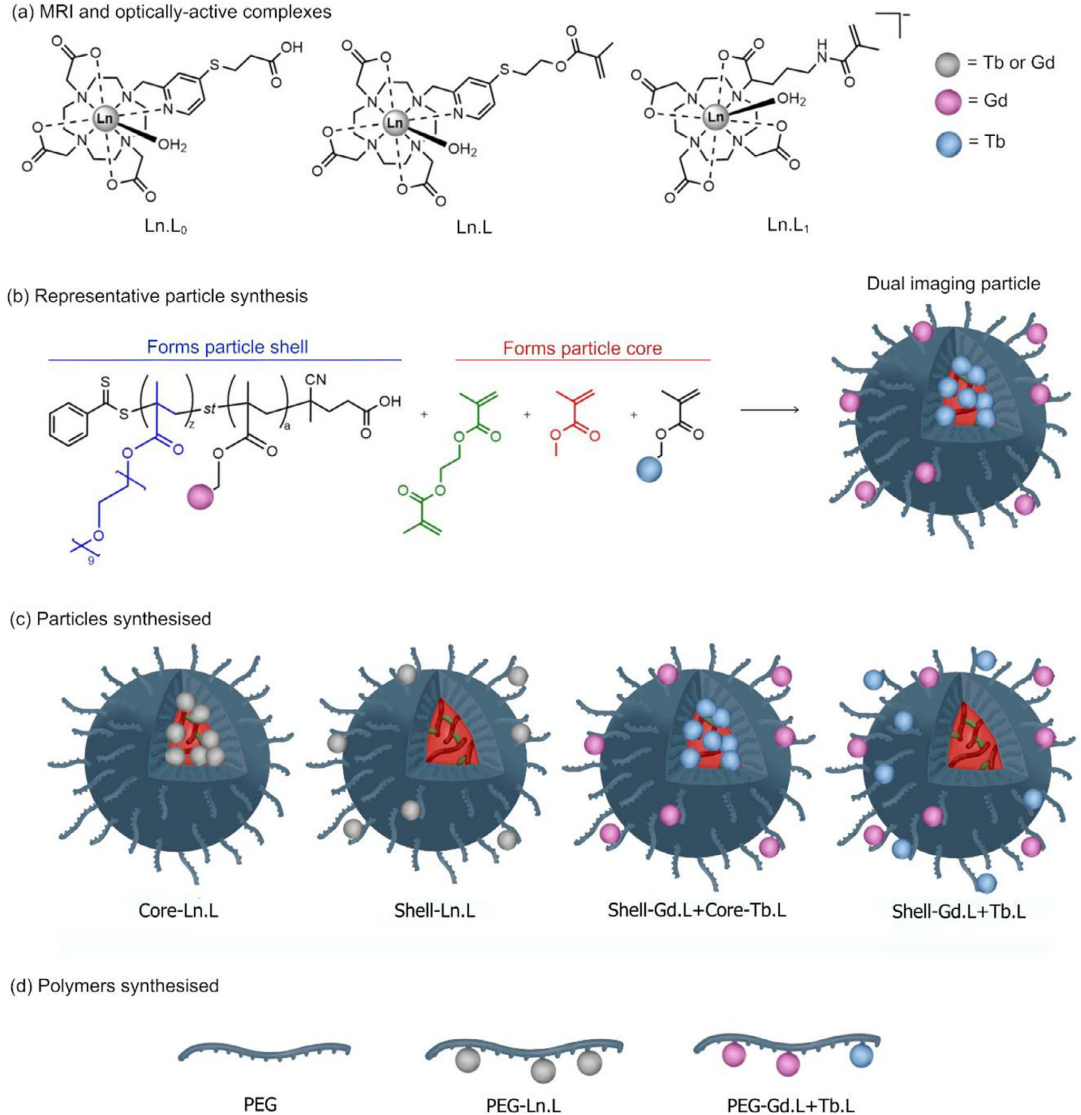
(a) Discrete lanthanide complexes of Tb(III) and Gd(III) used in this work, including the novel polymerizable complex **Ln.L**. (b) Schematic illustration of their incorporation into core–shell dual MRI–luminescence particles. (c) Core–shell particles incorporating Gd(III), Tb(III), or both, localized in either the core or shell. (d) Linear PEG‐based polymers containing **Gd.L**, **Tb.L,** or both. The linear polymer **PEG‐Gd.L_1_** and core‐shell particle **Shell‐Gd.L_1_**, each containing **Gd.L_1_** [38], were synthesised as reference systems for comparison with the **Ln.L**‐containing polymers.

Previously, we reported a novel method to effectively couple the global motion of hyperbranched polymers with the motion of the paramagnetic center. We synthesized macromolecular contrast agents via RAFT polymerization of stable Gd(III) monomers and crosslinkers, featuring a DOTA‐like core bearing one or two pendant methacrylamide arms [38]. Copolymerization of **Gd.L_1_**, bearing a single methacrylamide arm (Figure 1a), with *N*‐acryloylmorpholine (NAM) resulted in linear polymers with higher relaxivities (12.6–13.5 mM^−1^ s^−1^ at 60 MHz) and slower tumbling than the discrete complex **Gd.L_1_**. Hyperbranched polymers incorporating crosslinked Gd(III) chelates displayed even higher relaxivities (up to 22.8 mM^−1^ s^−1^ at 60 MHz) and slower tumbling due to the restricted motion of the crosslinked chelates, which doubled both the local and global reorientation correlation times, relative to the linear polymers containing **Gd.L_1_** [38].

To enable precise control over particle size and stability, we subsequently utilized RAFT emulsion polymerization to synthesize a library of amphiphilic core‐shell nanoparticles [39]. These particles feature a hydrophobic poly(methyl methacrylate) (p(MMA)) core crosslinked with ethylene glycol dimethacrylate (EGDMA) and are stabilized by a brush‐like hydrophilic p(OEGMEM) shell. MMA was selected due to its biocompatibility, hydrophobicity, and chemical stability, enabling robust core–shell particle formation while permitting sufficient water access for MRI contrast generation. EGDMA was used to provide structural stability to the particle core under aqueous conditions. Dynamic light scattering revealed stable particle diameters ranging from 33 to 176 nm with a low polydispersity index (PDI < 0.2). The use of p(OEGMEM) blocks enables control over the particle's hydrodynamic diameter (D_h_) and facilitates the incorporation of stable Gd(III) monomers into the shell, as demonstrated with **Gd.L_1_** [39].

With this background in mind, we set out to establish a simple and reproducible method to incorporate two complementary MRI and luminescence agents into well‐defined core–shell block copolymer particles (Figure 1). A cyclen‐based ligand scaffold bearing a single methacrylate group was designed, enabling the direct synthesis of isostructural Gd(III) and Tb(III) complexes, **Gd.L** and **Tb.L** (Figure 1a). This strategy ensured consistent polymerization behavior and direct incorporation of the complexes into block copolymers (Figure 1b). It also overcomes the drawbacks of traditional pendant‐group conjugation and post‐conjugation metalation [24, 27], including unreliable ligand loading, incomplete complex formation, and residual unchelated lanthanide ions, which must be avoided for clinical translation.

The novel complex **Ln.L** was incorporated into polymers to produce three distinct macromolecules (Figure 1c): the **Core‐Ln.L** particles, with the chelate located in the particle core; the **Shell‐Ln.L** particles, with the chelate positioned in the particle shell; and the brush‐like polymer **PEG‐Ln.L**. Crucially, the strategy presented here enables spatial control over the location of each imaging agent, positioning Gd(III) complexes in the hydrophilic shell to maximize relaxivity, while retaining flexibility in the placement of the luminescent Tb(III) component. By spatially separating the MRI‐active Gd(III) and optically active Tb(III) complexes within the same particle, we seek to tune the performance of each modality independently, thereby establishing a platform for future dual‐modal imaging applications. Particles incorporating both **Gd.L** and **Tb.L** into the shell achieved the highest relaxivity (*r*_1_ = 30.2 mM^−1^s^−1^ at 32 MHz, 298 K) and long‐lived Tb(III) luminescence, suitable for time‐gated removal of short‐lived background fluorescence in biological samples. These results confirm that the ligand design and spatial positioning of the Ln(III) complexes are critical for optimizing MRI and luminescence performance.

## 2 Results and Discussion

### 2.1 Design and Synthesis

The design of the polymerizable complex **Ln.L** aimed to combine high thermodynamic and kinetic stability with efficient lanthanide sensitization and compatibility with controlled RAFT polymerization. A cyclen‐based DO3A ligand scaffold (1,4,7,10‐tetraazacyclododecane‐1,4,7‐triacetic acid) was selected for its high thermodynamic and kinetic stability [40], and modified by incorporating a pyridyl pendant arm functionalized with a methacrylate group through a thioether linker, enabling direct polymerization. The 4‐substituted pyridine arm sensitizes Tb(III) luminescence [41, 42], while the Gd(III) analogue retains MRI suitability, as previously reported Gd(III) complexes bearing a single pyridine arm exhibit reasonably fast water‐exchange kinetics (*k*_ex_ approximately 2.7 × 10^6^ s^−1^, 298 K) [43]. This modular design permits the direct synthesis of both **Gd.L** and **Tb.L**, ensuring consistent polymerization behavior and precise incorporation of the complexes within core–shell block copolymer particles, thereby enabling dual MRI and luminescence capabilities.

**SCHEME 1:**
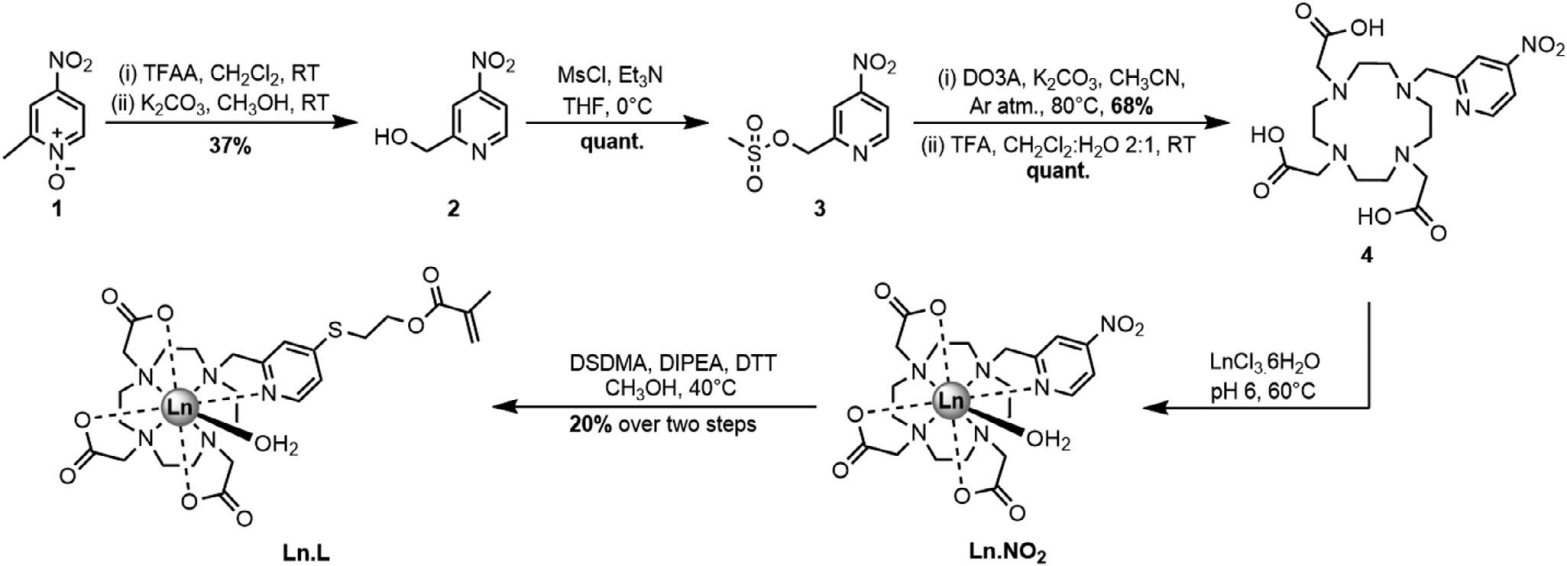
Synthesis of the polymerizable lanthanide(III) complex **Ln.L**. The methacrylate functionality was introduced at a late stage from the corresponding 4‐nitropyridine precursor complex **Ln.NO_2_**.

The synthesis of **Ln.L** (Ln = Tb and Gd) is described in Scheme 1 (full characterization provided in the ESI including Figures S1–S9). Briefly, *N*‐alkylation of 2‐methyl(sulfonyloxymethyl)‐4‐nitropyridine **3** onto the free secondary amine of DO3A(O*t*Bu)_3_ yielded the protected ligand following column chromatography. Removal of the *tert‐*butyl protecting groups using trifluoroacetic acid provided free ligand **4** in quantitative yield. The corresponding lanthanide complexes **Ln.NO_2_** were readily prepared by adding one equivalent of metal chloride salts, LnCl_3_ (Ln = Tb, Gd) to ligand **4** in water adjusted to pH 6. Finally, complexes **Ln.NO_2_** were reacted with bis(2‐methacyloyl)oxyethyl disulfide in the presence of DTT and *N,N*‐diisopropylethylamine to facilitate nucleophilic aromatic substitution of the 4‐nitropyridine group [41, 44] and formation of **Ln.L**, bearing a single methacrylate arm attached via a thioether bond. Complexes **Tb.L** and **Gd.L** were purified by reverse‐phase HPLC, ready for incorporation into polymeric particles. The control complex, **Ln.L_0_** (Figure 1a), bearing a non‐polymerizable thiother group, was prepared by reacting **Ln.NO_2_** with the water‐soluble 3‐mercaptopropionic acid in 5 mM HEPES buffer (pH 7) at 37°C. Finally, **Gd.L_1_**, the overall negatively charged Gd(III) complex bearing a single methacrylamide arm, was synthesized following our previously reported methodology [38].

### 2.2 Synthesis of Block Copolymer Particles Containing Ln.L and Gd.L_1_

To generate PEG‐based hydrophilic polymer shells, we adapted the protocol from our previous work [39]. The polymerization was conducted with an initial OEGMEM monomer concentration of 0.4 M, using 4‐cyano‐4‐(thiobenzoylthio)pentanoic acid (CTP) as the RAFT agent and 2,2′‐azobisisobutyronitrile (AIBN) as the initiator. The ratio of [CTP]/[AIBN] was maintained at 5. The ratio of CTP to monomer was 45 for the OEGMEM monomer, and 0.2 for the **Ln.L** monomer, aiming to produce p(OEGMEM)_45_‐*st*‐p(**Ln.L**)_0.2_], the **PEG‐Ln.L** precursor. The reaction was performed on a 10 mL scale using a mixture of water/DMSO (80/20). After 3 h, at around 90% conversion (kept below 95% to ensure end group fidelity) the solution was purified by dialysis and lyophilized to afford the target shell polymer as a viscous pink oil. In the next step, the initial chain was capped with additional OEGMEM to give [p(OEGMEM)_45_‐*st*‐p(**Ln.L**)_0.2_]‐b‐p(OEGMEM)_5_, which we refer to as **PEG‐Ln.L** (Figure 1d). This step prevented lanthanide complexes from localizing near the end of the p(OEGMEM) chains, which could interrupt particle self‐assembly or limit water access to **Gd.L** within the self‐assembled particles. The copolymerization was achieved using the same protocol as described for the first step, except using p(OEGMEM)_45_‐*st*‐p(**Ln.L**)_0.2_ as the RAFT agent. PEG‐**Ln.L** polymers were analyzed by gel permeation chromatography (GPC) to determine the molecular weight and dispersity of the generated brush‐like polymers (Table S3, Figures S8 and S9).

**FIGURE 2:**
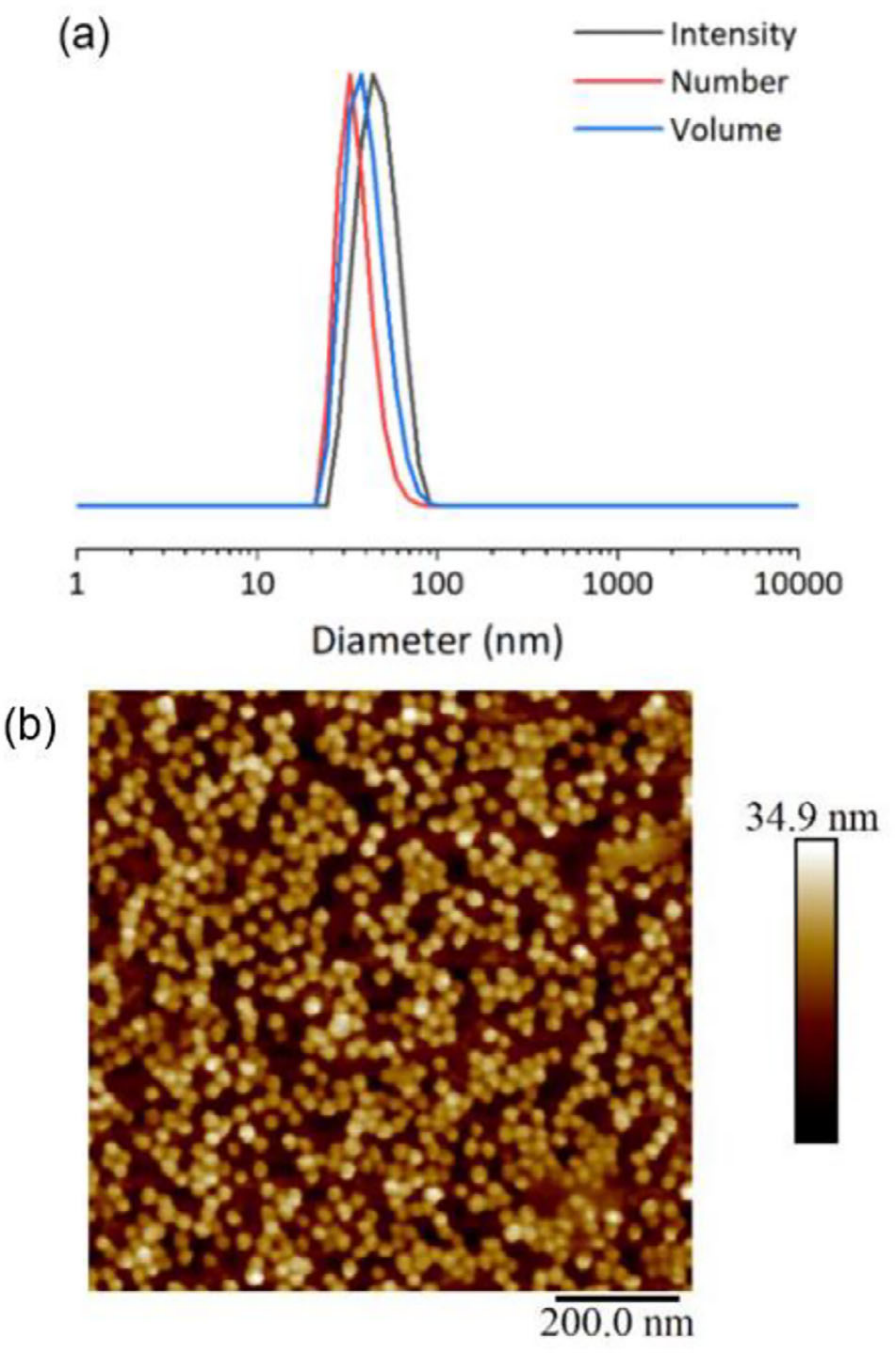
Particle size analysis of **Core‐Tb.L**. (a) DLS gave a z‐average diameter of 45 nm, with a PDI of 0.04. (b) AFM image showing spherical particles with an average diameter of 27 ± 3 nm (measured across 50 particles).

A RAFT‐emulsion polymerization was then used to synthesize nanoparticle **Shell‐Ln.L** [p(OEGMEM)_45_‐*st*‐p(**Ln.L**)_0.2_]‐*b*‐p(OEGMEM)_5_‐*b*‐[p(MMA)_250_‐*st*‐p(EGDMA)_4_]. MMA and EGDMA were selected to maintain consistency with our previously validated PMMA/p(OEGMEM) particles for potential MR imaging applications [39]. To achieve this, the precursor [p(OEGMEM)_45_‐*st*‐p(**Ln.L**)_0.2_]‐*b*‐p(OEGMEM)_5_ was stirred in water with 0.2 equivalents of AIBA, and MMA (250 equiv.) was added dropwise under continuous stirring. The solution was then purged and heated for 30 min, followed by the delayed addition of EGDMA crosslinker and **Ln.L**. After a further 1.5 h reaction, the mixture was purified by dialysis, giving the target core‐shell particles as a cloudy pink solution. This method yielded well‐defined (PDI ≈ 0.08), monodisperse particles with hydrodynamic diameters (D_h_) ranging from 19 to 115 nm, which exhibited minimal aggregation and remained stable for 6 months (Table S4, Figures S10–S18). Atomic force microscopy (AFM) images of the dried particles revealed diameters ranging from 14 to 69 nm (Figure 2 and Figure S19). ICP‐MS analysis confirmed effective incorporation of the Ln(III) complexes into the particles, with an average loading efficiency of 46% at high conversion (Tables S5 and S6). The timing of **Ln.L** and **Gd.L_1_** complex addition was varied, either during shell formation or during core formation, to determine their final positions within the particles (Scheme S1).

### 2.3 Photophysical Analysis of Tb.L and Tb.L‐Polymers

Photophysical analysis of the monomeric complex **Tb.L,** and the corresponding polymeric particles incorporating **Tb.L**, is summarized in Table 1. The UV‐vis absorption spectrum of **Tb.L** in aqueous solution (pH 7.0, 295 K) displays a broad band with a maximum at 278 nm, assigned to the pyridyl group bearing an electron‐donating thioether at the 4‐position (Figure S20). The extinction coefficient (*ε*) of **Tb.L** was determined to be 14,735 M^−1^cm^−1^ (Figure S21). Excitation of this pyridine antenna resulted in characteristic terbium(III) emission in the green region of the visible spectrum between 475–675 nm (Figure S21), with no detectable ligand fluorescence at shorter wavelengths, consistent with efficient energy transfer from the pyridyl antenna to the metal center. A quantum yield of 7.3% was determined relative to quinine sulfate [45]. The emission lifetime of **Tb.L** increases from 1.74 ms in H_2_O to 2.62 ms in D_2_O, consistent with reduced non‐radiative quenching by O–D oscillators compared to O–H and enabling determination of a hydration number, *q*, of 0.66 [46]. This indicated the presence of one coordinated water molecule, with the non‐integer value likely reflecting an equilibrium between mono‐aqua species and minor populations containing more weakly bound solvent, influenced by steric effects of the pyridyl pendant arm [46].

**TABLE 1:** Photophysical analysis of Tb.L and Tb.L‐containing particles.

Sample	λ_max_/nm	τ_H2O_/ms	τ_D2O_/ms	*q* ^b^
Tb.L ^a^	278	1.74	2.62	0.66
PEG‐Tb.L	282	1.52	2.30	0.81
Shell‐Tb.L	282	1.56	2.23	0.65
Core‐Tb.L	282	1.56	2.26	0.69
PEG‐Tb.L+Gd.L	282	1.52	2.30	0.82
Shell‐Tb.L+Gd.L	282	1.68	2.57	0.73
Core‐Tb.L+Shell‐Gd.L	282	1.33	1.84	0.74

^a^ Extinction coefficient (*ε*) of **Tb.L** = 14,735 M^−1^cm^−1^, quantum yield, Φ_em_, of **Tb.L** = 7.3%, measured using quinine sulfate in 0.05 M H_2_SO_4_ (Φ_em_ = 60%) as standard [45].

^b^ Hydration state, *q*, was determined from the emission lifetime measurements in H_2_O and D_2_O following literature methods, with an estimated error of ± 20% [46].

**FIGURE 3:**
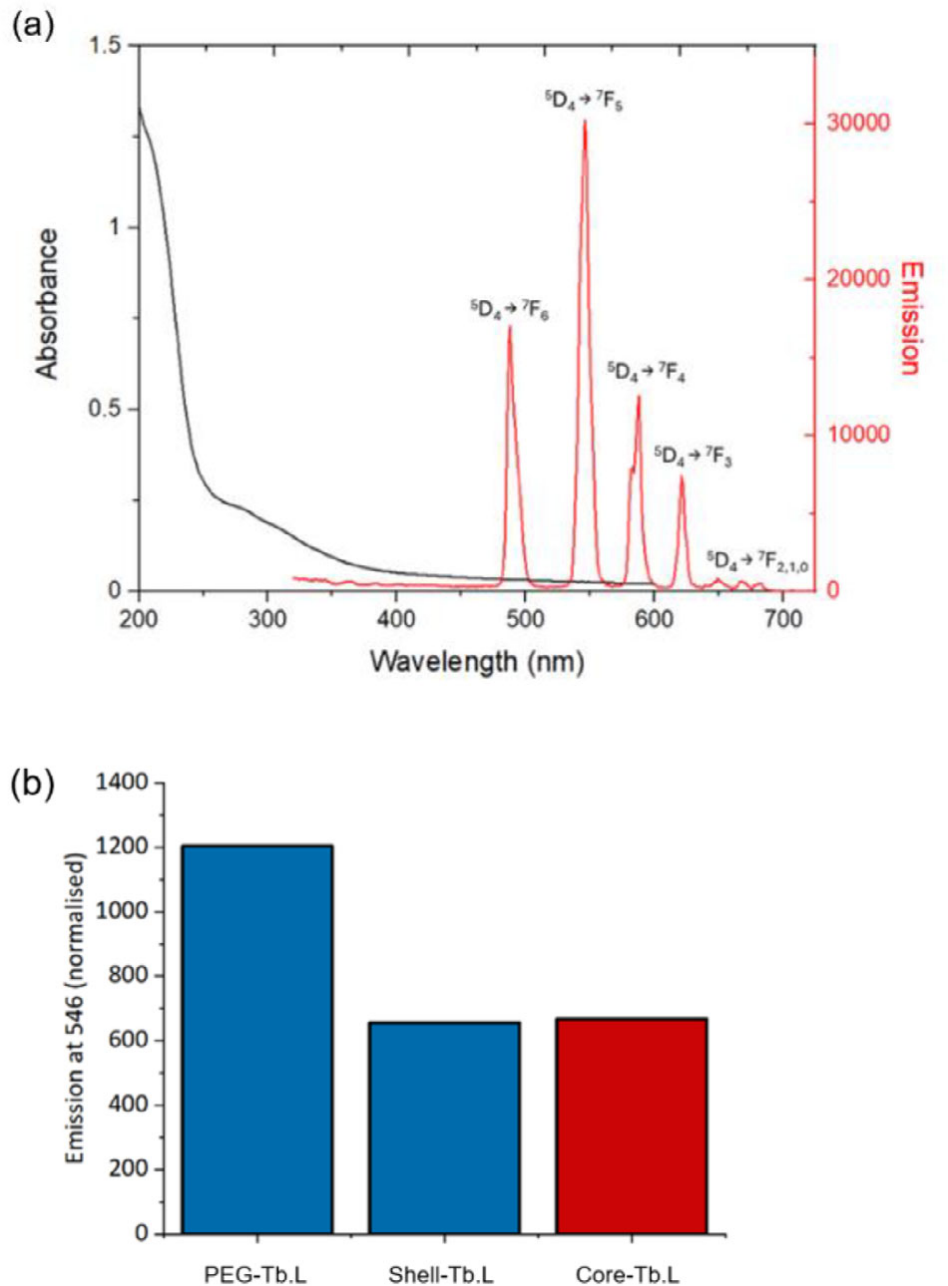
(a) Absorbance (black) and time‐resolved emission (red) spectra of **Shell‐Tb.L** in water (pH 7.0, 295 K). The electronic transitions corresponding to emission bands are indicated. (b) Comparison of emission intensity at 546 nm for **Tb.L**‐containing polymers following 10‐fold dilution in water (pH 7.0, 295K). Emission intensities are normalized to the Tb(III) concentrations determined by ICP‐MS‐calculated Tb(III) concentrations.

For the purified linear polymers and core–shell particles, containing **Tb.L,** the UV–vis spectra showed a peak at 282 nm (Figure 3 and Figures S20 and S22), confirming successful incorporation of **Tb.L**, consistent with ICP–MS data (Figure S1). The slight red shift from 278 to 282 nm is attributed to a change in the pyridine electronic structure induced by polymerization of the peripheral methacrylamide group.

An additional lower energy absorption band at 305 nm arises from thioester chain‐transfer agent (CTA) end groups (Figure S22), with higher relative intensity in the brush‐like polymers **PEG‐Tb.L** and **PEG‐Tb.L+Gd.L** compared with the core‐shell particles, possibly reflecting the location of the chain ends within the particle core. Upon excitation at 282 nm, the polymer particles exhibit the expected line‐like Tb(III) emission (Figure 3 and Figures S23–S25). Some non‐uniform background fluorescence (350–475 nm) is observed in the steady‐state emission spectra, attributed to light scattering from the polymer matrix. This background fluorescence was effectively removed by time‐resolved emission spectroscopy, using a 60 µs delay (Figure 3a). This isolates the characteristic Tb(III) luminescence and highlights the advantage of long‐lived Tb(III) emission for improving signal‐to‐noise in complex aqueous environments.

Extinction coefficient and absolute quantum yield determination for the polymeric systems proved challenging due to the overlapping UV band that prevented accurate quantitation. Nonetheless, following normalization of the **Tb.L** concentration using the ICP–MS data (Table S5), comparable emission intensities for **Core‐Tb.L** and **Shell‐Tb.L** were observed (Figure 3b). Because Tb(III) emission is relatively insensitive to changes in hydration, this does not necessarily reflect similar water environments; indeed, the Gd‐based relaxivity data (vide infra) indicates clearly distinct hydration states in the core and shell regions. By contrast, the linear **PEG‐Tb.L** displayed nearly double the emission intensity relative to the particles, suggesting that the architecture and steric environment of the particle systems partially quenches Tb(III) emission.

Emission lifetime measurements (Figures S26–S28) of the polymeric systems in H_2_O and D_2_O yielded values approximately 13% lower than for the discrete complex **Tb.L,** with calculated *q* values between 0.65 and 0.82, consistent with approximately one coordinated water molecule in all systems (Table 1). Notably, **Core‐Tb.L** and **Shell‐Tb.L** exhibited identical lifetimes (1.56 ms in water) and *q* values, supporting the notion that the Tb(III) coordination environment remains similar irrespective of its location within the particle.

### 2.4 Relaxivity of Discrete Gd(III) Complexes

**FIGURE 4:**
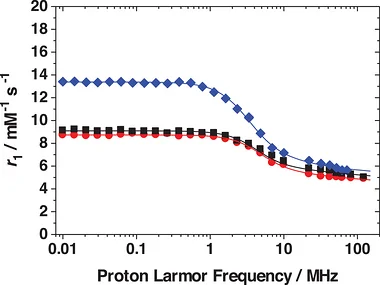
NMRD profiles of discrete complexes **Gd.L_1_** (Blue); **Gd.L** (Black); **Gd.L_0_** (Red), measured in water at 298K.

Nuclear magnetic dispersion (NMRD) profiles were measured at three different temperatures (283, 298, and 310 K) for the monomeric complexes **Gd.L** and **Gd.L_0_** and compared to the previously reported data for **Gd.L_1_** (Figure 4 and Figure S29). In the high field region, the relaxivities are similar for the three complexes, as their comparable molecular weight and size results in similar rotational dynamics. The data were fitted using established paramagnetic relaxation theory referred by the Solomon–Bloembergen–Morgan (SBM) and Freed's equations for the inner (IS) and outer‐sphere (OS) proton relaxation mechanisms, respectively (Table 2, see ESI for details) [47]. Detailed information on the water‐exchange kinetics was obtained by measuring the temperature dependence of the ^17^O NMR transverse relaxation rate (*R*_2_) and paramagnetic shift (Δω) (Figure S30). The data for **Gd.L_0_** were collected at 14.1 T and pH 7.4 and analyzed according to the well‐established set of Swift–Connick equations [48]. The number of coordinated water molecules was fixed at *q* = 1, based on the emission lifetimes of the Tb(III) analogues in water and D_2_O, together with the structural similarities to previously reported mono‐pyridyl Gd(III) complexes [43].

**TABLE 2:** Relaxometric parameters for **Gd.L_0_** and **Gd.L** obtained from ^17^O NMR (**Gd.L_0_**) and ^1^H NMRD data. **Gd.L_1_** parameters included for comparison [38].

Fitting parameters^a^	Gd.L_0_	Gd.L	Gd.L_1_
*r_1_* at 298/310 K, mM^−1^ s^−1^	5.1/4.2	5.7/4.8	6.5/5.1
τ_M_/µs	1.2 ± 0.1	1.2^b^	0.154
ΔH^‡^/kJ mol^−1^	55.5 ± 0.2	55.5^b^	43.8
^298^τ_RG_/ps	98 ± 1	115 ± 2	106
$\tau_{V}^{298}$/ps	27.8 ± 1.5	28.0 ± 1.1	32.5
*Δ* ^2^/10^19^ s^−2^	2.4 ± 0.1	2.2 ± 0.2	0.94
*Q*	1	1	1

^a^ The following parameters were fixed during the fitting procedure: the hydration number *q* was set to 1; distance between Gd(III) and bound water protons, r, was set to 3.0 Å; distance of closest approach of outer sphere water molecules to Gd(III) (a)= 4.0 Å; relative diffusion coefficient D^298^ = 2.25 × 10^−5^ cm^2^ s^−1^ with activation energy E_D_ = 20 kJ mol^−1^; and activation energy of τ_V_ (E_V_) = 1 kJ mol^−1^.

^b^ Values were determined for **Gd.L_0_** and considered unchanged for the fitting the NMRD profiles of **Gd.L**.

The rotational correlation times of **Gd.L_0_** and **Gd.L** were determined to be 98 and 115 ps, respectively, in line with the slightly higher molecular weight of **Gd.L**. The rate of water exchange is almost 8‐times lower for **Gd.L_0_** with respect to **Gd.L_1_** and, thus, represents the parameter most influenced by the nature of the donor groups. Accordingly, the enthalpy of activation is higher. For Gd(III) chelates, replacement of negatively charged carboxylate donors with neutral amide donors typically slows water exchange due to lower steric hindrance, decreased electron density at the metal center and reduced overall negative charge of the complexes [49]. Similarly, substituting one carboxylate donor group with a neutral pyridine donor appears to have a comparable effect, even though steric hindrance is not reduced. The negative charge on **Gd.L_1_** is known to increase the water exchange rate by inducing slight repulsion between the bound water molecule and the complex. As expected, due to their increased molecular weight, each Gd(III) complex in this study has a higher relaxivity than that of Gd.DOTA (Dotarem) under similar conditions (4.7 mmol^−1^ s^−1^, 20 MHz, 298 K).

### 2.5 Relaxivity of Gd.L and Gd.L_1_ in Particles

Each macromolecule containing **Gd.L** or **Gd.L_1_** was analysed by ^1^H NMR relaxometry at varying temperatures and magnetic fields, summarized in Table 3. For comparison, two polymeric systems incorporating the previously reported complex **Gd.L_1_** [38] were also synthesized: the linear **PEG‐Gd.L_1_** polymer and the core‐shell **Shell‐Gd.L_1_** particle. Relaxivity values determined at 32 MHz and 298 K were 18.2 mM^−1^s^−1^ for the linear polymer **PEG‐Gd.L**, lower than that of the analogous **PEG‐Gd.L_1_** (24.0 mM^−1^s^−1^, Figure S31). The core‐shell particles, **Shell‐Gd.L** and **Core‐Gd.L** exhibited *r*_1_ values of 23.7 and 18.0 mM^−1^s^−1^, respectively. The substantial increases in relaxivity of the macromolecular **Gd.L** and **Gd.L_1_** systems compared with the discrete **Gd.L** and **GdL_1_** complexes (Table 2) confirm that the chelates were successfully incorporated into the polymeric architectures. Similarly, **Shell‐Gd.L_1_** and **Core‐Gd.L_1_** gave relaxivity values of 25.8 and 17.6 mM^−1^s^−1^, respectively. The higher relaxivities of the shell‐located chelates (**Shell‐Gd.L** and **Shell‐Gd.L_1_**) compared to the core‐located chelates (**Core‐Gd.L** and **Core‐Gd.L_1_**) likely reflects greater second‐ and outer‐sphere water interactions in the particle shell, whereas restricted access of bulk water to the Gd(III) centers in the particle core limits relaxivity.

**TABLE 3:** NMRD data of polymers containing **Gd.L_1_** or **Gd.L,** and dual MRI–Luminescence particles containing **Gd.L** and **Tb.L**.

Fitting Parameters^a^	Polymers with GdL_1_		Polymers with Gd.L			Dual imaging systems	
	PEG‐Gd.L_1_	Shell‐Gd.L_1_	PEG‐Gd.L	Shell‐Gd.L	Core‐Gd.L	PEG‐Gd.L+Tb.L	Shell‐Gd.L+Tb.L
*r_1_* at 298/310K 32 MHz /mM^−1^ s^−1^	24.1/21.0	25.8/26.7	18.2/19.3	23.7/25.5	18.0/20.5	16.0/17.0	30.2/33.0
^298^τ_RG_/ns	2.5 ± 0.1	4.0 ± 0.2	2.8 ± 0.2	3.8 ± 0.1	4.1 ± 0.2	2.7 ± 0.2	4.1 ± 0.2
^298^τ_RL_/ns	0.37 ± 0.03	0.34 ± 0.05	0.22 ± 0.01	0.44 ± 0.03	0.20± 0.02	0.21 ± 0.03	0.38 ± 0.03
S^2^	0.23 ± 0.02	0.35 ± 0.02	0.28 ± 0.02	0.41 ± 0.01	0.28 ± 0.01	0.24 ±0.02	0.42 ± 0.01
^298^τ_v_/ps	57 ± 1	10 ± 1	44 ± 2	17 ± 1	32 ± 2	42 ± 1	15 ± 2
*Δ* ^2^/10^19^ s^−2^	0.29 ± 0.05	0.54 ± 0.07	0.63 ± 0.07	0.41 ± 0.07	0.43 ± 0.05	0.78 ± 0.07	0.55 ± 0.08
Q	1	1	1	1	1	1	1
^298^τ_M_/µs	0.33^b^	0.33^b^	0.60 ± 0.07^c^	0.50 ± 0.06^c^	0.50	0.6 ± 0.1^c^	0.50 ± 0.05^c^
*Δ*H^‡^/kJ mol^−1^	—	—	13.6 ± 0.8^c^	14 ± 1^c^	—	12.4 ± 0.7^c^	16 ± 1^c^

^a^ To fit the ^1^H NMRD data at 298 K, the following parameters were fixed: *q* = 1, r_Gd‐H_ 3.0 A, a_Gd‐H_ = 4 A, ^298^D_Gd‐H_ = 2.25 × 10^5^ cm^2^ s^−1^.

^b^ Values reported in Berki et al. [38].

^c^ Estimated from VT NMR relaxivity profiles (Figure S33) at fixed magnetic field (20 MHz).

**FIGURE 5:**
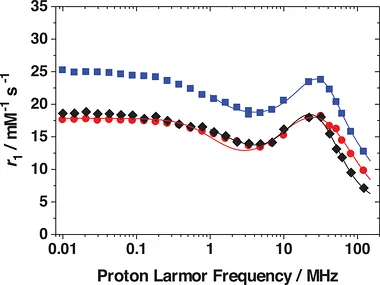
NMRD profiles of the **Gd.L**‐containing macromolecules **Shell‐Gd.L** (blue); **Core‐Gd.L** (black); **PEG‐Gd.L** (red). Variable temperature data shown in Figure S33.

NMRD profiles of the polymeric systems (PEG, shell and core) generally showed maxima at 20–30 MHz, consistent with slow rotational correlation times typical of macromolecules (Figure 5 and Figures S31–S34). One outlier, **Core‐Gd.L_1_**, lacked a peak in this region (Figure S32), suggesting poor incorporation of this complex. ICP‐MS confirmed that **Core‐Gd.L_1_** had the lowest Gd(III) loading (Table S7, 0.07 mM vs. 0.32 mM for **Core‐Gd.L**), reflecting the reduced compatibility of the more hydrophilic and charged **Gd.L_1_** complex with the hydrophobic particle core. In addition to its greater hydrophilicity, the methacrylamide group of **Gd.L_1_** may be less reactive with methyl methacrylate monomers than the methacrylate group in **Gd.L**, reducing covalent incorporation. For this reason, **Core‐Gd.L_1_** was not included in the fitting procedure.

For the macromolecular systems, fitting was performed using the model‐free Lipari–Szabo approach incorporated into the Solomon–Bloembergen–Morgan (SBM) equations [50]. This model accounts for (i) the global rotational correlation time (τ_RG_) of the entire macromolecule; (ii) the local correlation time (τ_RL_) of the Gd(III) complex; and (iii) the order parameter (S^2^), which reflects the degree of coupling between global and local motions (S^2^ = 1 indicates full coupling, S^2^ = 0 no coupling). As shown in Table 3, among the macromolecules incorporating the novel complex **Gd.L**, **Shell‐Gd.L** exhibited the highest relaxivity (23.7 mM^−1^s^−1^ at 298K), attributed to a combination of slow tumbling (τ_RG_ = 3.8 ns) and more effective coupling of local and global motion (S^2^ = 0.41), possibly due to the dense packing of PEG side chains in the particle shell. However, the molecular‐level origin of this motion restriction cannot be unequivocally resolved from the current experimental data and could benefit from molecular dynamics simulations to be fully elucidated. In contrast, linear **PEG‐Gd.L** and **Core‐Gd.L** showed lower S^2^ values (0.28), indicating that **Gd.L** mobility was less restricted in these systems. Between the linear brush‐like polymers, **PEG‐Gd.L_1_** exhibited higher relaxivity than **PEG‐Gd.L** (24.1 and 18.2 mM^−1^s^−1^ respectively). Despite the higher molecular weight of **PEG‐Gd.L_1_** (61.7 vs 22.1 kDa), τ_RG_ values were comparable (2.5 vs 2.8 ns), suggesting that the increased relaxivity of **PEG‐Gd.L_1_** arises from the faster water exchange rate of **Gd.L_1_** compared with **Gd.L** (Table 2). A similar trend was observed for the particles, where **Shell‐Gd.L_1_** exhibited higher relaxivity than **Shell‐Gd.L** (25.8 vs 23.7 mM^−1^s^−1^, respectively), consistent with the faster water exchange of **Gd.L_1_** when incorporated into the particle shell.

The significantly higher relaxivity of **Shell‐Gd.L** relative to **Core‐Gd.L** confirms the importance of spatial positioning of the Gd(III) complex within the particle and highlights the potential for stimulus‐responsive systems that modulate water access to the core (e.g., via pH variation). Notably, **Shell‐Gd.L** achieved a high relaxivity (23.7 mM^−1^s^−1^) using a simple monofunctional Gd(III) complex that can be synthesized in just six steps from commercial materials with installation of a single polymerizable group. The particle architecture provides a defined spherical shape and distinct environments: a shell accessible to water and a core that can be protected or exploited for responsive systems. Positioning the Gd(III) complexes in the particle shell maximized relaxivity (23.7 and 25.8 mM^−1^ s^−1^ for **Gd.L** and **Gd.L_1_**, respectively), whereas core localization gave lower values (18.0 and 17.6 mM^−1^ s^−1^), due to restricted water access in the hydrophobic core.

### 2.6 Dual MRI–Luminescence Polymers and Particles

The ultimate goal of this work was to develop particles with optimized positioning of the MRI and luminescence agents. Optimizing the block copolymer formulation and systematically incorporating either Gd(III) or Tb(III) complexes revealed three key findings: (1) stable particles with small diameters were consistently obtained using a p(OEGMEM)_50_ stabilizing block and a target DP of 250 for the MMA core; (2) the position of **Tb.L** in the shell or core had minimal influence on the emission intensity or lifetime (Table 1); and (3) positioning **Gd.L** in the particle shell resulted in significantly enhanced relaxivity, compared with the core‐localized analogue (Table 3).

Based on these results, dual MRI‐luminescence agents were prepared with **Gd.L** positioned in the shell to maximize MRI contrast, while **Tb.L** was located either in the shell or in the core, giving two distinct architectures: **Shell‐Gd.L+Tb.L** and **Core‐Tb.L+Shell‐Gd.L** (Figure 1c). In addition, we prepared the brush‐like polymer **PEG‐Gd.L+Tb.L** (Figure 1d) to enable direct comparison with **PEG‐Gd.L**. The linear polymer **PEG‐Gd.L+Tb.L** showed a slightly lower relaxivity than the analogous **PEG‐Gd.L** (16.0 and 18.2 mM^−1^ s^−1^ at 298 K and 32 MHz, see Table 3). Relaxation measurements at 60 MHz (Table S8) indicate that the incorporation of **Tb.L** complexes within the dual particles has no substantial influence on the observed relaxivity.

**FIGURE 6:**
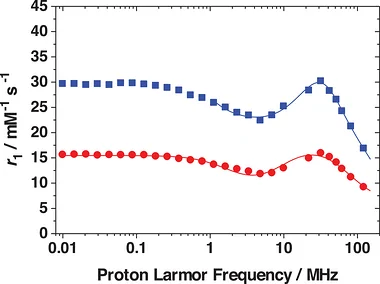
NMRD profiles for the dual MRI–luminescence particles **Shell‐Gd.L+Tb.L** (blue) and **PEG‐Gd.L+Tb.L** (red).

The dual MRI–luminescence particles exhibit characteristic NMRD profiles for large macromolecules (Figure 6 and Figure S34), similar to those obtained for **Gd.L**‐containing macromolecules. **Shell‐Gd.L+Tb.L**, containing both Tb(III) and Gd(III) complexes in the particle shell, gave the highest relaxivity of all macromolecules studied in this work (30.2 mM^−1^s^−1^, Table 3). The relaxivity obtained here approaches the highest from our prior work, where a value of 32.7 mM^−1^s^−1^ was achieved using a Gd(III) complex bearing two *cis*‐polymerizable arms to form a p(NAM)‐based hyperbranched polymer. In that system, the *cis‐*oriented arms immobilized the complex, effectively coupling it to the global tumbling of the polymer (S^2^ = 0.6) [38]. By contrast, the current design achieves comparably high relaxivity without the need for extensive synthesis of crosslinking chelates. Notably, the dual imaging particle **Shell‐Gd.L+Tb.L** achieved more than a 6‐fold increase in relaxivity at 310K compared with the discrete **Gd.L** complex. Surprisingly, the dual imaging particle with **Tb.L** located in the core (**Shell‐Gd.L+Core‐Tb.L**) showed markedly lower relaxivity (15 mM^−1^ s^−1^, Figure S34d). Although the origin of this reduced relaxivity is not fully understood, the result highlights the influence of spatial positioning of the two agents on the MRI performance. Importantly, a consistent trend is observed in the S^2^ values across the dual imaging systems: **Shell‐Gd.L+Tb.L** exhibits a comparable S^2^ value (0.42) to **Shell‐Gd.L** and a higher value than the corresponding linear **PEG‐Gd.L+Tb.L** system (0.24), indicating that the restricted Gd(III) local motion within the particle shell is preserved upon incorporation of Tb(III) complexes.

Finally, to confirm that the Gd(III)‐based and the dual Gd(III)/Tb(III) nanoparticles discussed in this work function as positive contrast agents, the transverse longitudinal relaxivity (*r*_2_) was also measured at 21 MHz and 298 K for **Shell‐Gd.L** and **Shell‐Gd.L+Tb.L**, giving values of 34.2 mM^−1^ s^−1^ and 38.4 mM^−1^ s^−1^, respectively. In both cases, the *r*_2_/*r*_1_ ratio was lower than 2 (1.47 for **Shell‐Gd.L** and 1.33 for **Shell‐Gd.L+Tb.L**) confirming that the nanoparticles predominantly shorten the longitudinal relaxation time, therefore producing a brighter signal on *T*_1_‐weighted MRI images.

The presence of **Tb.L** in the dual MRI–luminescence particles resulted in the characteristic line‐like terbium(III) emission upon indirect excitation at 278 nm (Figure S35). Both the emission intensity and lifetime of **Shell–Gd·L+Core–Tb·L** were approximately 20% lower than those of **Shell–Gd·L+Tb·L** (Figure S36 and Table 1), reflecting the relative insensitivity of Tb(III) emission to changes in local hydration, as Tb(III) is less affected by O–H vibrational quenching compared with, for example, Eu(III). The dual‐imaging particles exhibited *q* values between 0.73 and 0.82, consistent with one coordinated water molecule in all systems. Finally, the optical stability of the Tb(III)‐containing particles was evaluated across pH 3–10, showing no significant changes in either particle size or emission intensity (Figure S37). This pH insensitivity is promising for potential biological applications, indicating that the luminescent output remains robust under physiological pH variations.

## 3 Conclusion

In summary, we have developed a modular polymeric platform with dual MRI–luminescence properties, by incorporating Gd(III) and Tb(III) complexes into core–shell block copolymer particles using a common macrocyclic ligand bearing a single methacrylate group. This ligand design streamlined the synthesis and enabled similar polymerization kinetics for both complexes, while enabling precise spatial positioning of the MRI and luminescence components. Relaxometric studies demonstrated that positioning the Gd(III) complex in the hydrophilic shell consistently produced higher relaxivities compared with the core‐localized analogues, reflecting improved water accessibility and stronger coupling between local and global rotational dynamics.

**FIGURE 7:**
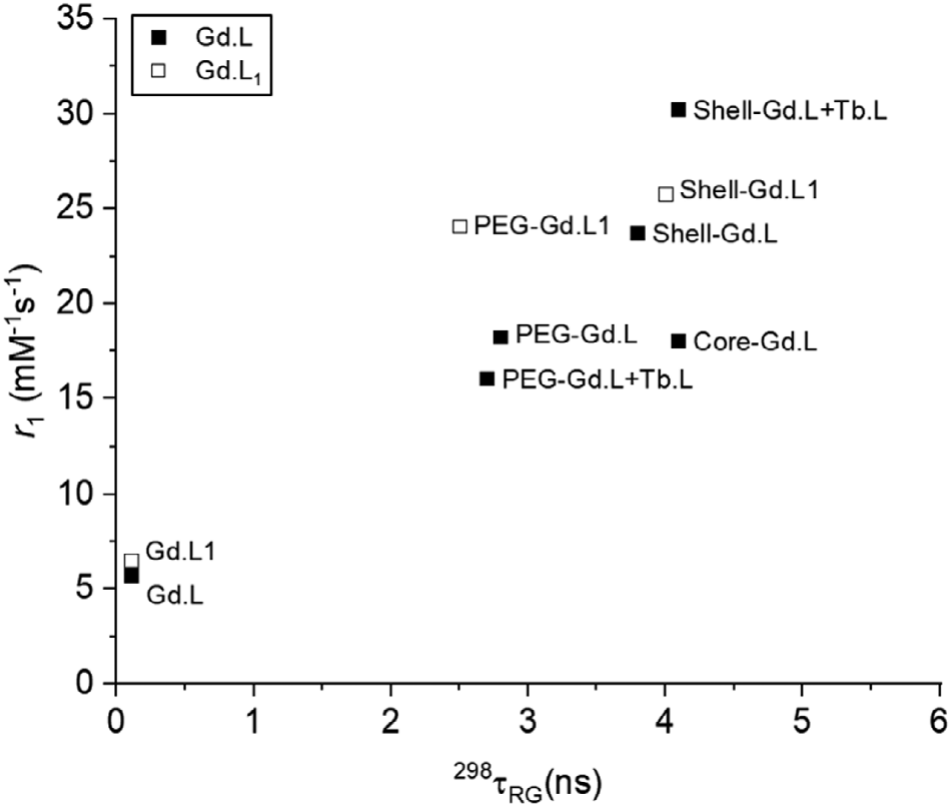
Plot of global rotational correlation time, ^298^τ_RG_, against the corresponding relaxivity, *r*_1_, for **Gd.L**‐containing systems (black boxes) and those containing **Gd.L_1_** (white boxes).

As shown in Figure 7, an approximately linear relationship between global rotational correlation time and relaxivity is evident for polymeric systems containing **Gd.L_1_** (red boxes), consistent with its relatively fast water exchange rate. **Gd.L_1_** systems generally showed slightly higher relaxivities than those containing **Gd.L**, likely due to faster water exchange, although its negative charge limited incorporation into the hydrophobic particle core. For the dual‐imaging systems containing **Gd.L** and **Tb.L**, while the brush‐like polymers **PEG‐Gd.L+Tb.L** and **PEG‐Gd.L** show similar relaxivities, their incorporation into core‐shell particles substantially increased both *τ_RG_* and relaxivity (Table S9). For example, chain extension of **PEG‐Gd.L+Tb.L** with MMA to form **Shell‐Gd.L+Tb.L** almost doubled the relaxivity, from 16.0 to 30.2 mM^−1^ s^−1^. This value is over 5 times higher than the discrete **Gd.L** complex and surpasses values reported for many linear polymers and dendrimers [51, 52, 53, 54, 55, 56, 57]. Indeed, our particles achieve relaxivities that rival those of systems exploiting biological structures (e.g., 29.7 mM^−1^ s^−1^) [58] and matches recent high‐relaxivity polymeric systems, such as crosslinked chitosan nanogels incorporating bifunctional Gd‐DOTAGA_2_ chelates (30 mM^−1^ s^−1^ at 30 MHz, 298 K) [59, 60].

This performance illustrates how precise spatial positioning of Gd(III) and Tb(III) complexes within core–shell particles, enabled by a common ligand design, can be used to tune relaxivity and luminescence contrast. Together, these features provide a versatile platform for developing dual‐modal MRI–luminescence particles with high relaxivity and potential for stimuli‐responsive imaging applications. Dedicated dual‐modal imaging studies remain an important focus for future work to evaluate performance in biological environments. Further investigation of the biological behavior of these PEGylated nanoparticles, including biodistribution and clearance, will also be necessary to assess their suitability for in vivo imaging applications.

## Conflicts of Interest

The authors declare no conflict of interest.

## Supporting information




**Supporting File**: smll74389‐sup‐0001‐SuppMat.docx.

## Data Availability

The data that supports the findings of this study are available in the supplementary material of this article.
