# Supplementary material for: A Modular Core–Shell Nanoparticle Platform for Dual‐Modal MRI–Luminescence With High Relaxivity
Source: Small. 2026 Jul 3;22(48):e74389. doi: 10.1002/smll.74389 (PMC13509071; doi:10.1002/smll.74389)
Supplement: Supplementary file 1 [file SMLL-22-e74389-s001.docx]

Supplementary Information

**A Modular Core–Shell Nanoparticle Platform for Dual-Modal MRI–Luminescence with High Relaxivity**

Catherine J. Ashton^ab^, Jonathan Martinelli^c^, Sara Camorali^c^, Thomas R. Berki^ab^, Amy J. Managh^a^, Constantina Sofroniou^b^, Lorenzo Tei^c^, Helen Willcock*****^b^ and Stephen Butler*^a^

^a^Department of Chemistry, Loughborough University, Leicestershire, LE11 3TU, United Kingdom.

^b^Department of Materials, Loughborough University, Leicestershire, LE11 3TU, United Kingdom.

^c^Department of Science and Technological Innovation, Università del Piemonte Orientale, I15121, Alessandria, Italy.

*Correspondence: S.J.Butler@lboro.ac.uk; H.Willcock@lboro.ac.uk

Contents

[1.	Materials and Methods	3](#_Toc215412240)

[General Considerations	3](#_Toc215412241)

[Nuclear Magnetic Resonance	3](#_Toc215412242)

[Liquid Chromatography Mass Spectrometry	4](#_Toc215412243)

[Column Chromatography	4](#_Toc215412244)

[High Performance Liquid Chromatography	4](#_Toc215412245)

[Luminescence Experiments	4](#_Toc215412246)

[ICP-MS Measurements	5](#_Toc215412247)

[GPC	6](#_Toc215412248)

[DLS	6](#_Toc215412249)

[AFM	7](#_Toc215412250)

[2.	Synthesis and Characterisation of Ligands and Corresponding Ln(III) Complexes	7](#_Toc215412251)

[Tri-tert-butyl-2,2’,2”-(1,4,7,10-tetraazacyclododecane-1,4,7-triyl)-triacetate (DO3A)	7](#_Toc215412252)

[2-Hydroxymethyl-4-nitropyridine (2)	8](#_Toc215412253)

[Methyl-m-3-nitropyridine sulfonate methyl ester (3)	9](#_Toc215412254)

[1,4,7,10-Tetraazacyclododecane-1-(methyl-m-3-nitropyridine)-4,7,10-tris(t-butyl acetate) (Protected Ligand 4)	10](#_Toc215412255)

[1,4,7,10-Tetraazacyclododecane-1-(methyl-m-3-nitropyridine)-4,7,10-triacetic acid (4)	11](#_Toc215412256)

[Terbium(III) (1,4,7,10-Tetraazacyclododecane-1-(methyl-m-3-nitropyridine)-4,7,10-triacetic acid) (Tb.NO_2_)	12](#_Toc215412257)

[Gadolinium(III) (1,4,7,10-Tetraazacyclododecane-1-(methyl-m-3-nitropyridine)-4,7,10-triacetic acid) (Gd.NO_2_)	13](#_Toc215412258)

[**Complex Tb.L**	14](#_Toc215412259)

[**Complex Gd.L**	15](#_Toc215412260)

[**Complex Tb.L_0_:** Terbium(III) (1,4,7,10-Tetraazacyclododecane-1-(methyl-m-3-[mercaptopropionic acid])-4,7,10-triacetic acid)	16](#_Toc215412261)

[**Complex Gd.L_0_**: Gadolinium(III) (1,4,7,10-Tetraazacyclododecane-1-(methyl-m-3-[mercaptopropionic acid])-4,7,10-triacetic acid)	17](#_Toc215412262)

[3.	Synthesis of Macromolecular Imaging Agents	18](#_Toc215412263)

[Synthesis of block copolymer particles containing Ln.L	18](#_Toc215412264)

[3.1 Copolymerisation of OEGMEM and **Gd.L** to generate **PEG-Gd.L** precursor	18](#_Toc215412265)

[3.2 Chain Extension with OEGMEM to generate **PEG-Gd.L**	19](#_Toc215412266)

[4.	Characterisation of Ln.L-Containing Macromolecules	22](#_Toc215412267)

[GPC Analysis of Ln.L-containing Macromolecules	22](#_Toc215412268)

[DLS Analysis of Ln.L-containing Macromolecules	23](#_Toc215412269)

[ICP-MS Analysis	28](#_Toc215412270)

[UV-Vis Analysis of Tb.L and Tb.L-containing Macromolecules	29](#_Toc215412271)

[Emission Spectra of Ln.L-containing Macromolecules	30](#_Toc215412272)

[Emission Decay Curves of Tb.L-containing Macromolecules in H_2_O and D_2_O	32](#_Toc215412273)

[Relaxivity Analysis	33](#_Toc215412274)

[Appendix	39](#_Toc215412275)

[Representative ^1^H-NMR Spectra of Particles	49](#_Toc215412276)

[References	51](#_Toc215412277)

## 1. Materials and Methods

### General Considerations

Reagent grade chemicals, including the anhydrous solvents, were purchased from Sigma Aldrich and Fluorochem and used without further purification.

### Nuclear Magnetic Resonance

^1^H, ^13^C, COSY, HMQC and HMBC NMR spectra were recorded in the stated deuterated solvent on a JEOL ECS-400 or 500 spectrometers (^1^H at 400 MHz, ^13^C at 101 MHz; ^1^H at 500 MHz, ^13^C at 126 MHz, respectively), at 298 K. Chemical shifts are expressed in ppm and are adjusted to the chemical shift of the residual NMR solvent resonances (CDCl_3_: ^1^H δ = 7.26 ppm, ^13^C δ = 77.16 ppm, CD_3_OD: ^1^H δ = 3.31 ppm, ^13^C δ = 49.00 ppm or DMSO-*d_6_*: ^1^H δ = 2.50 ppm, ^13^C δ = 39.52 ppm, D_2_O: ^1^H δ = 4.80 ppm). The coupling constants are expressed in Hz.

Polymer samples were made up of 50 µL reaction mixture, and 6.5 mL of deuterated solvent. Trioxane was added as a reference to each reaction mixture (5.1 ppm). This allowed tracking of the disappearance of monomer methacrylate vinyl hydrogen peaks (∼6.0 and 5.6 ppm) by comparison of the peak integration values to that of trioxane. This method was used to track all polymerisations conducted, RAFT and RAFT-emulsion. NMR tracking of the RAFT polymerisations was conducted in D_2_O. The tracking of emulsion polymerisations was conducted in CDCl_3_, a small sample of the aqueous reaction mixture (50 µL) was shaken with 0.6 mL CDCl_3_. An aqueous layer containing the particles sat at the top of the NMR sample. It was assumed that unreacted monomer was present in the deuterated chloroform layer and so was visible by NMR. Both crosslinked and un-crosslinked emulsion polymerisations were tracked in this manner. NMR was also used to calculate degree of polymerisation, as described in our previous work.^[1]^

### Liquid Chromatography Mass Spectrometry

Liquid Chromatography Electrospray Mass spectra were recorded on a Shimadzu Prominence LC system with a Shimadzu SPDM20A Photodiode Array Detector, a Shimazu CTO-20A column oven, Shimadzu SIL-20A autosampler and a Shimadzu LCMS 20 mass spectrometer controlled using LabSolutions software. The system operates in positive ion mode, with acetonitrile as the carrier solvent. The flow rate was maintained at 0.7 mL/min over a gradient of 5 to 95% acetonitrile or methanol in water (0.1% formic acid) for 10 minutes. High resolution mass spectra were recorded using a Thermofisher Q-Exactive orbitrap mass spectrometer.

### Column Chromatography

Column chromatography was performed using flash silica gel 60 (particle size 40–63 microns) purchased from Apollo Scientific. Thin layer chromatography (TLC) was performed on aluminium sheet silica gel plates with 0.2 mm thick silica gel 60 F254 using the stated mobile phase.

### High Performance Liquid Chromatography

Preparative RP-HPLC was performed using a Waters 2489 UV/Visible detector performed at 254 nm, a Waters 1525 Binary HPLC pump controlled by the Waters Breeze 2 HPLC system software. Separation was achieved using a semi-preparative XBridge C18 (5 μm OBD 19 × 100 mm) column at a flow rate maintained at 17 mL/min). A solvent system composed of either water (0.1% formic acid)/acetonitrile (0.05% formic acid) was used over the stated linear gradient. Analytical RP-HPLC was performed using a XBridge C18 5 μm 4.6 × 100 mm at a flow rate maintained at 2.0 mL/min using the same gradients and solvents.

### Luminescence Experiments

Luminescence spectra were recorded on a Camlin Photonics luminescence spectrometer with FluoroSENS version 3.4.7.2024 software. Emission spectra were obtained using a 40 µL or 100 µL Hellma Analytics quartz cuvettes. Excitation light was set at the absorption maxima and emission recorded in the range 400 – 720 nm using an integration time of 0.5 seconds, increment of 1.0 nm, excitation slit of 0.2 nm and emission slit of 0.5 nm. Quantum yields were measured using quinine sulfate in 0.05 M H_2_SO_4_ as a standard (Φ_em_= 0.60, λ_ex_ = 350 nm).^[2]^ Emission lifetime measurements were performed on the FluoroSENS instrument. Measurements were taken of 1 mL of 0.1 absorbance samples of Tb(III) complexes in 10 mM HEPES at pH 7.0, unless stated otherwise. Measurements were obtained by indirect excitation of the Tb(III) ion via the pyridine antenna using a short pulse of light at the absorbance λ_max_ of the antenna (typically ~283 nm), followed by monitoring the integrated intensity of the light emitted at 615 nm, with 500 data points collected over a 10 millisecond time period. The decay curves were plotted in Origin Labs 2019 version 9.6.0.172, and fitted to the equation:

$$I = A0 + A1 e^{-kt}$$

where I is the intensity at time, t, following excitation, A_0_ is the intensity when decay has ceased, A_1_ is the pre-exponential factor and k is the rate constant for the depopulation of the excited state.

The hydration state, *q*, of the Tb(III) complexes was determined using the modified Horrocks equation^[3]^:

$$q (Tb) = 5 ( \frac{1}{\tau H2O}-\frac{1}{\tau D2O} - 0.06)$$

where $\tau$_H2O_ and $\tau$_D2O_ are the observed emission lifetimes in water and D_2_O, respectively.

Time-resolved emission spectra of the Tb(III)-containing polymers were recorded from 350 to 720 nm in 1 nm steps, using a gated detection window scanned from 60 to 400 µs following excitation.

### ICP-MS Measurements

The first step of this analysis was the formation of Tb(III) and Gd(III) standards, using a multi-element stock solution with a concentration of 10,000 ppb which contained both lanthanides. This was useful as it meant that only one set of standards was required. This stock solution was diluted by a factor of 10 to give a stock solution of 1000 ppb.

| **Table S1.** Dilution of multi-element stock solution. | | | | | |
| --- | --- | --- | --- | --- | --- |
| Stock Solution | Starting Conc. | Aliquot | Total Volume | Dilution Factor | Final Conc. |
|  | (ppb) | (mL) | (mL) |  | (ppb) |
| Multi-element | 10000 | 1 | 10 | 10 | 1000 |

Once the 1000 ppb stock solution was generated, calibration standards were generated, containing differing amounts of the multi-element stock solution.

| **Table S2.** Formulations for the production of ICP-MS calibration solutions. | | | | | | |
| --- | --- | --- | --- | --- | --- | --- |
| Standard Number | Component | Tb(III) + Gd(III)  Multi-element *^a^* | 2 % Nitric acid *^b^* | Total Volume | |  |
| 1 | Volume Added / ml | 0 | 9.50 | 10 | |  |
|  | Final Conc. / ppb | 0 |  |  | |  |
| 2 | Volume Added / ml | 0.05 | 9.45 | 10 | |  |
|  | Final Conc. / ppb | 5 |  |  | |  |
| 3 | Volume Added / ml | 0.1 | 9.40 | 10 | |  |
|  | Final Conc. / ppb | 10 |  |  | |  |
| 4 | Volume Added / ml | 0.2 | 9.30 | 10 | |  |
|  | Final Conc. / ppb | 20 |  |  | |  |
| 5 | Volume Added / ml | 0.4 | 9.10 | 10 | |  |
|  | Final Conc. / ppb | 40 |  |  | |  |
| 6 | Volume Added / ml | 0.6 | 8.9 | 10 | |  |
|  | Final Conc. / ppb | 60 |  |  | |  |
| ^a^ Multi-element solution containing Tb(III) and Gd(III) at a concentration of 1000 ppb. ^b^ A 2% nitric acid solution was used to dilute the calibration standards up to the total volume of 10 mL. | | | | |  |  |

Production of the calibration standards allowed generation of a calibration graph, relating the known concentrations of the Tb(III) and Gd(III) standards to a given Tb(III) or Gd(III) signal (cps) produced by the ICP-MS instrument, Figure S1. The two calibration graphs show a very similar trend due to the use of a multielement stock solution which had the same concentration of both Gd(III) and Tb(III).

**Figure S1.** Calibration graph of Tb(III) standards produced for ICP-MS.

The calibration plot fitting gave the equation:

Tb(III) Signal = 907398 × Tb(III) ppb

With an R^2^ = 0.9896. This equation was used to relate the signal from the ICP-MS samples to a given concentration.

Following digestion in 67% nitric acid, the particle samples were diluted 3500-fold to an estimated Ln(III) concentration suitable for the ICP-MS instrument. Hence, to obtain the initial concentrations of the particle sample, the “ICP-MS Sample” ppb values were multiplied by 3500 to give the “undiluted sample” ppb, and then converted to mM. The Gd.L-only particles were not included in this analysis as these samples were analysed by ICP-MS alongside the relaxivity measurements conducted at Università del Piemonte Orientale.

### GPC

Molecular weight and dispersity analysis were performed using Agilent Technologies 1260 infinity GPC, with either chloroform/triethylamine (RI) or THF (universal calibration) as the mobile phase and polystyrene standards. Raw data was accessed using Agilent GPC/SEC software. Universal analyses measurements were performed on an Agilent Infinity 1260 MDS instrument, equipment with refractive index (RI) and viscometry detectors (VS). The system was equipped with 2 Agilent PLGel Mixed C analytical columns and an Agilent PLGel 5 µm guard. The system was run in THF with 0.1% BHT (butylated hydroxy toluene) at 30 °C at 1 ml/min. A universal calibration was created from narrow polystyrene standards (Agilent EasiVials) between 2,210,000 and 540 g/mol. Analysis was performed using Agilent GPC/SEC software.

### DLS

DLS measurements were performed using a Malvern Zetasizer Nano NS and Zetasizer software, version 7.12. The system uses an avalanche photodiode detector and a diode-pumped solid-state laser operating (wavelength of 532 nm). Light scattered was detected at a 175° angle, and the temperature was stabilised at 25 °C ± 0.1 °C. Particle suspensions were diluted 10 µL in 1 mL deionised water and filtered through 400 nm nylon syringe filters.

### AFM

AFM topography images were obtained with a Bruker BioScope Resolve. Measurements were performed using tapping mode and silicon cantilevers (RTESPA-150) with typical spring constants of 5 N/m and tip radius of 8 nm. Samples were diluted ten-fold with deionised water, cast onto glass slides, and allowed to dry at room temperature. The resulting images were analysed using ImageJ software. 50 Particle diameter measurements were taken from each image. Care was taken to measure equal numbers of particles in each quadrant of the image, to avoid clusters of similarly sized particles. The raw data was analysed to give a mean and standard error.

## 2. Synthesis and Characterisation of Ligands and Corresponding Ln(III) Complexes

### Tri-tert-butyl-2,2’,2”-(1,4,7,10-tetraazacyclododecane-1,4,7-triyl)-triacetate (DO3A)

Under a nitrogen atmosphere and over a period of 2 hours, *tert*- butylbromoacetate (5.06 mL, 34.3 mmol, 2.95 equiv.) was added dropwise to an ice-cold stirring solution of cyclen (2.00 g, 11.6 mmol, 1 equiv.) and sodium hydrogen carbonate (2.93 g, 34.8 mmol, 3 equiv.) in anhydrous acetonitrile (40.0 mL). The reaction was then stirred at room temperature for 3 days, before the solids were removed via filtration and the solvent evaporated. The remaining residue was purified by flash column chromatography (silica gel, pure CH_2_Cl_2_ to 97:3 CH_2_Cl_2_/CH_3_OH with an increment of 1%) to give **DO3A** (1.89 g, 3.6 mmol, 32%) as a white solid. **IR (*v_max_*/cm^-1^, neat):** 2977, 2851, 2733, 1716, 1367, 1254, 1145, 848. **^1^H NMR (500.16 MHz, CDCl_3_)** *δ* **ppm:** 1.43(2) (9H, s, H^1^), 1.43(9) (18H, s, H^10^), 2.85(0) (4H, m, H^5^), 2.99(4) (8H, m, H^6,11^), 3.08 (4H, m, H^12^), 3.27 (2H, s, H^4^), 3.35 (4H, s, H^7^), 10.02 (1H, s, NH). **^13^C NMR (126 MHz, CDCl_3_)** *δ* **ppm:** 170.6 (C^8^) 169.7 (C^3^), 81.9 (C^3^), 81.7 (C^9^), 58.3 (C^7^), 51.4, 51.3, 49.3, 47.6 (C^5, 6, 11, 12^), 48.9 (C^4^), 28.3(0) (C^1^), 28.2(6) (C^10^). ***R*_f_ (CH_2_Cl_2_/CH_3_OH 90:10 v/v):** 0.52. **HRMS (ESI^+^, m/z):** calculated for [M+H^+^]: 515.3803, found 515.3804 g/mol.

### 2-Hydroxymethyl-4-nitropyridine (2)

4-Nitro-2-picoline N-oxide **1** (2.00 g, 13.0 mmol) was dissolved in CH_2_Cl_2_ (40 mL), before the addition of trifluoroacetic anhydride (5.5 g, 26.2 mmol) in CH­_2_Cl_2_ (6.0 mL). After stirring at room temperature for 4 days, another equivalent of trifluoroacetic anhydride was added, resulting in completion of the reactions after another 2 days. The solvent was removed under vacuum to give an oil, which was dissolved in methanol (20 mL). A saturated solution of potassium carbonate was added to the reaction mixture until pH 8. The solution was stirred for 18 hours, before the solvent was removed and the dry oil dissolved in ethyl acetate (85 mL) and washed with brine (3 x 150 mL). Although in future it would be recommended that the oil be dissolved in brine, and extracted with ethyl acetate, to prevent loss of product into the aqueous layer. The organic layer was dried over magnesium sulphate and the solvent removed under vacuum to give compound **2** as a dark orange solid (0.74 g, 37%). **IR (*v_max_*/cm^-1^, neat):** 3225, 1670, 1537, 1204, 1177, 1121, 833, 721. ***R*_f_ (**CH_2_Cl_2_/CH_3_OH 99:1 v/v): 0.65. **^1^H NMR (500.16 MHz, DMSO)** *δ* **ppm:** 4.71 (2H, d, ^3^J_H-H_ = 5.9Hz, H^6^), 5.79 (1H, t, ^3^J_H-H_ = 5.9Hz, O-H), 7.99 (1H, ddt, ^3^J_H-H_ = 5.4Hz, ^4^J_H-H_ = 2.3Hz, Ar2), 8.10 (1H, m, ^4^J_H-H_ = 1.6Hz, Ar4), 8.87 (1H, dd, ^3^J_H-H_ = 5.4Hz, Ar1). **^13^C NMR (126 MHz, CDCl_3_)** *δ* **ppm:** 63.6 (C6); 112.2 (C4); 114.5 (C2); 151.4 (C1); 154.4 (C3, q); 165.9 (C5, q). Spectral data were in accordance with that reported in the literature (K. L. Gempf *et al*).^[4]^

### Methyl-m-3-nitropyridine sulfonate methyl ester (3)

2-Hydroxymethyl-4-nitropyridine **2** (275 mg, 1.79 mmol) was dissolved in anhydrous THF (20 mL) under inert conditions. Triethylamine (0.74 mL, 5.32 mmol) was added, followed by methane sulphonyl chloride (0.17 mL, 2.14 mmol) and the reaction mixture stirred for 15 minutes at 0^°^C, before the solvent was removed under reduced pressure. The residue was dissolved in dichloromethane (38 mL) and washed with brine (38 mL). The aqueous layer was extracted into chloroform (2 x 28 mL) and the organic layers were combined and dried over magnesium sulphate and concentrated under reduced pressure to give **3** (420 mg, quant.) as an orange oil. The mesylate ester **3** was taken to the next reaction immediately. **^1^H NMR (500.16 MHz, CDCl_3_)** *δ* **(ppm):** 3.18 (3H, s, H^7^), 5.46 (2H, s, H^6^), 8.03 (1H, dd, ^3^J_H-H_ = 5.4Hz, ^4^J_H-H_ = 2.1Hz, Ar2), 8.20 (1H, d, ^4^J_H-H_ = 2.1Hz, Ar4), 8.92 (1H, d, ^3^J_H-H_ = 5.4Hz, Ar1). **^13^C NMR (126 MHz, CDCl_3_**) *δ* **ppm:** 38.1 (C7); 69.6 (C6); 114.6 (Ar5); 116.1 (Ar3); 151.9 (Ar6); 154.6 (Ar2); 157.4 (Ar4). **LC-MS (ESI^+^, m/z):** calculated for C_7_H_9_N_2_O_5_S [M+H^+^]: 233.0230, found 232 g/mol. Spectral data were in accordance with that reported in the literature (K. L. Gempf *et al*).^[4]^

### 1,4,7,10-Tetraazacyclododecane-1-(methyl-m-3-nitropyridine)-4,7,10-tris(t-butyl acetate) (Protected Ligand 4)

Mesylate ester **3** (420 mg, 1.81 mmol), the macrocyclic triester, DO3A*^t^*Bu (825 mg, 1.6 mmol), and potassium carbonate (247 mg, 1.79 mmol) were dissolved in anhydrous acetonitrile (18 mL), under an atmosphere of argon with stirring. The mixture was accidentally heated to ~150 °C for 2 minutes, before being allowed to cool for 15 minutes. Solids were removed from the reaction mixture via centrifugation and the solvent removed under reduced pressure. The crude material was purified using alumina gel chromatography (neat CH_2_Cl_2_ to 5% CH_3_OH/CH_2_Cl_2_ v/v), to remove the four-arm (DO4A) impurity giving the protected version of ligand **4** (708 mg, 68%) as a dark orange oil. **IR (***v_max_***/cm^-1^, neat):** 1/λ 2977, 2936, 2827 (alkane C-H stretch), 1723 (O=C-O-R stretch), 1155 (aliphatic ester stretch). ***R*_f_** (CH_2_Cl_2_/CH_3_OH 95:5 v/v): 0.325. **^1^H NMR (400 MHz, CDCl_3_)** *δ* **ppm:** 1.25 (27H, m, 3 x *^t^*Bu), 1.93-4.10 (24H, m, macrocyclic Hs (16) + 4 x CH_2_), 7.77 (1H, dd, ^3^J_H-H_ = 5.4Hz, ^4^J_H-H_ = 1.8Hz, Ar2), 7.86 (1H, d, ^4^J_H-H_ = 1.8Hz, Ar4), 8.43 (1H, d, ^3^J_H-H_ = 5.4Hz, Ar1). **^13^C NMR (400 MHz, CDCl_3_)** *δ* **ppm:** 28-82 (28.0, 28.0, 27.9, 55.5, 55.7, 59.0, 82.0, 82.2, CH_2_ and CH_3_, 114.8 (C4), 116.0 (C2), 150.9 (C1), 154.3 (C3), 163.0 (C5), 172.9, 173 (C10). **HRMS (ESI^+^, m/z):** calculated for [M+Na^+^]: 673.3895, seen 673.3893 g/mol, calculated for [M+H]^+^: 651.4076, seen 651.4304 g/mol. **m.p.:** 185-188 °C. Spectral data were in accordance with that reported in the literature (K. L. Gempf *et al*).^[4]^

### 1,4,7,10-Tetraazacyclododecane-1-(methyl-m-3-nitropyridine)-4,7,10-triacetic acid (4)

*tert*-Butyl protected intermediate (319 mg, 0.299 mmol) was dissolved in CH_2_Cl_2_ (0.8 mL) and water (0.4 mL) added. The reaction mixture was cooled in an ice bath, and trifluoroacetic acid (3.9 mL) added slowly, before being stirred for 24 hrs at room temperature. The solvents were removed under reduced pressure, with repeated additions of dichloromethane (10 × 10 mL), to aid removal of trifluoroacetic acid, giving the ligand **4** (322 mg, quant, residual TFA remained) as a brown oil. The deprotected ligand **4** was used directly in the following complexation step. **IR** (*v_max_*/cm^-1^, neat): 3090 (sp2 C-H stretch), 2969, 2923, 2869 (alkane C-H stretch), 1718, 1670 (O=C-OH stretch). ***R*_f_** (CH_2_Cl_2_/CH_3_OH 50:50 v/v, 2 drops of ammonia): 0.74. **^1^H NMR (400MHz, D_2_O)** *δ* **ppm:** 3.18 – 3.64 (22H, m, macrocyclic and 3 x CH_2_), 8.14 (1H, dd, ^3^J_H-H_ = 5.6 Hz, ^4^J_H-H_ = 2.1 Hz, Ar2), 8.25 (1H, s, Ar4), 8.81 (1H, d, ^3^J_H-H_ = 5.6 Hz, Ar1). **HRMS (ESI^+^, m/z):** calculated for [M+H^+^] C_20_H_30_N_6_O_8_: 438.1075, seen 438.2198 g/mol. **m.p.:** 124 °C.

### Terbium(III) (1,4,7,10-Tetraazacyclododecane-1-(methyl-m-3-nitropyridine)-4,7,10-triacetic acid) (Tb.NO_2_)

The deprotected ligand **4** (144 mg, 0.3 mmol) was dissolved in water (4 mL) and the pH adjusted to pH 6 with sodium hydroxide solution (1 M). Terbium(III) chloride hexahydrate (125 mg, 0.34 mmol) was dissolved in water (4 mL) before being added to the reaction mixture, the pH was readjusted to pH 6, which caused precipitation of the complex. The reaction mixture was heated to 60 ^°^C for 16 h. The water was removed via lyophilisation, to give complex **Tb.NO_2_** (quant., TFA salt remained) as a pale orange solid. **IR** (*v_max_*/cm^-1^, neat): 1/λ 3117 (sp2 C-H stretch), 2994, 2928, 2879 (alkane C-H stretch), 1676, 1593 **(O=C-O-Tb stretch**). ***R*_f_** (CH_2_Cl_2_/CH_3_OH, 50:50 v/v): 0.06. **^1^H NMR (500.16 MHz, D_2_O)** *δ* **ppm:** 286.14, 257.64, 246.79, 240.22, 213.73, 184.71, 167.82, 160.50, 17.43, 4.79, 1.53, -11.58, -26.97, -32.78, -36.56, -37.82, -53.97, -57.02, -78.70, -97.88, -174.30, -194.77, -316.62, -337.83. **HRMS (ESI^+^, m/z):** calculated for [M+Na^+^]: 661.1036, seen: 661.1036 g/mol. **Mp:** decomposed at 237.8 ^º^C. **HPLC** [H2O (0.1% v/v HCOOH)/CH3CN(0.1% v/v HCOOH)]: **t_R_** = 11.172. **λ_max_:** 295 nm. **τ_H2O_:** 1.59 ms (0.1 ABS). **τ_D2O_:** 2.27 ms (0.1 ABS). ***q*:** 1 (0.63). **Φ:** 0.4 (average across three samples in H_2_O), 0.2 (in CH_3_OH).

**Figure S2.** Analytical HPLC trace of **Tb.NO_2_** moving from 100% acidified water to 100% acetonitrile over 23 minutes.

### Gadolinium(III) (1,4,7,10-Tetraazacyclododecane-1-(methyl-m-3-nitropyridine)-4,7,10-triacetic acid) (Gd.NO_2_)

The deprotected ligand **4** (98 mg, 0.2 mmol) was dissolved in water (4.5 mL) and the pH adjusted to pH 6 with sodium hydroxide solution (1 M). Gadolinium(III) chloride hexahydrate (82 mg, 0.22 mmol) was dissolved in water (1.5 mL) before being added to the reaction mixture, and the pH was readjusted to pH 6, which caused precipitation of the complex. The reaction mixture was heated to 60^°^C for 16 h. The water was removed via lyophilisation, to give complex **Gd.NO_2_** (quant., TFA salt remained) as a pale orange solid. **IR** (*v_max_*/cm^-1^, neat): 2286, 1677, 1596, 1396, 1182, 1125, 800, 721.). ***R*_f_** (CH_2_Cl_2_/CH_3_OH 50:50 v/v, 2 drops of ammonia): 0.35. **HRMS (ESI^+^, m/z):** calculated for [M+Na^+^]: 660.1024, seen 660.1023 g/mol. **HPLC:** [H2O (0.1% v/v HCOOH)/CH3OH(0.1% v/v HCOOH)]: **t_R_** = 3.014.

**Figure S3.** Analytical HPLC trace of **Gd.NO_2_** moving from 100% acidified water to 100% acidified methanol over 10 minutes.

### **Complex Tb.L**

The crude complex **Tb.NO_2_** (77 mg, est. 0.08 mmol, 1 eq.), bis(2-methacryloyl)oxyethyl disulfide (13 µL, 0.052 mmol, 0.65 eq.), and N,N-diisopropylethyl amine (27 µL, 0.154 mmol, 1.9 eq.) were dissolved in CH_3_OH (10 mL) and stirred at 30 ^°^C for 0.5 h. Dithiothreitol (2.5 mg, 0.016 mmol, 0.2 eq.) was then added and the reaction stirred for 24 h, before CH_3_OH was evaporated under reduced pressure. The crude product was then purified via HPLC to give complex **Tb.L** (12 mg, 20% over 2 steps) as a white powder. **IR (*v_max_*/cm^-1^, neat):** 3361, 2864, 1588, 1385, 1157, 1082, 716, 443. **R_f_ ( CH_2_Cl_2_/CH_3_OH, v/v 50:50):** 0.14. **^1^H NMR (500MHz, D_2_O):** 269, 258, 233, 218, 202, 191, 95, 91, 53, 18, 8, 0.2, -3, -8, -21, -39, -46, -50, -60, -70, -78, -95, -108, -126, -140, -144, -148, -166, -206, -321, -348, -354, -390. **HRMS-ESI: [M+H^+^]:** Calculated: 738.1611, seen: 738.1610, [M+Na^+^]: Calculated: 760.1420, seen: 760.1428, [M+K^+^]: Calculated: 776.1170, seen: 776.1167. **Mp:** decomposed at 245.8 ^º^C. **HPLC** [H2O (0.1% v/v HCOOH)/CH3CN(0.1% v/v HCOOH)]: t_R_ = 11.78 minutes. **Ɛ:** 14.735 ± 0.030 M^-1^cm^-1^. **λ_max_:** 278 nm. **τ_H2O_:** 1.74 ms (average across 3 samples, 0.01 ABS). **τ_D2O_:** 2.62 ms (average across 3 samples, 0.01 ABS). ***q*:** 1 (0.66). **Φ:** 9.9 (average across 3 samples in H_2_O), 11.0 (in CH_3_OH).

**Tb.L**

**Tb.NO_2_**

**Figure S4.** Analytical RP-HPLC traces showing a reproducible shift in retention time upon conversion of **Tb.NO₂** to **Tb.L**. Elution was performed using water (0.05% formic acid) and acetonitrile (0.05% formic acid) with a gradient of 0–50% acetonitrile over 5 minutes, followed by 50–75% over the next 10 minutes, and 75–100% over the following 3 minutes.

### **Complex Gd.L**

The crude complex **Gd.NO_2_** (81 mg, estimated 0.078 mmol, 1 equiv.), Bis(2-methacryloyl)oxyethyl disulfide (11 µL, 0.043 mmol, 0.55 eq), and N,N-diisopropylethyl amine (16 µL, 0.094 mmol, 1.2 eq) were dissolved in CH_3_OH (10 mL) and stirred at 30 ^°^C for 0.5 h. Dithiothreitol (3.63 mg, 0.024 mmol, 0.3 eq) was then added and the reaction stirred for 24 h, before CH_3_OH was evaporated under reduced pressure. The crude product was then purified via HPLC to give complex **Gd.L** (12 mg, 20% over 2 steps) as a white powder. **IR (*v_max_*/cm^-1^, neat):** 3344, 2857, 1584, 1383, 1082, 716, 435. ***R*_f_** (CH_2_Cl_2_/CH_3_OH, v/v 50:50): 0.11. **HRMS-ESI:** calculated for [M+Na^+^] C_26_H_36_N_5_O_8_SGd: 759.1418, seen 759.1420. **Mp:** decomposed at 249.3 ^º^C, **HPLC** [H2O (0.1% v/v HCOOH)/CH3CN(0.1% v/v HCOOH)]: **t_R_** = 11.779 minutes. ***Ɛ*:** 14.793 ± 0.053 M^-1^cm^-1^. **λ_max_:** 280 nm.

**Figure S5.** Analytical HPLC trace of **Gd.L** moving from 100% acidified water to 100% acidified methanol in stages to optimise separation. Elution was performed using water (0.05% formic acid) and methanol (0.05% formic acid) with a gradient of 0–50% methanol over 5 minutes, followed by 50–75% over the next 10 minutes, and 75–100% over the following 3 minutes.

### **Complex Tb.L_0_:** Terbium(III) (1,4,7,10-Tetraazacyclododecane-1-(methyl-m-3-[mercaptopropionic acid])-4,7,10-triacetic acid)

Complex **Tb.NO_2_** (~50 mg, estimated 0.05 mmol, 1 equiv.) was dissolved in HEPES solution (5 mM, 6.66 mL). 3-Mercaptopropionic acid (14 µL, 0.16 mmol) was added and the solution was stirred at 37 ^°^C for 18 h. The pH was regularly adjusted to pH 7 with the use of sodium hydroxide solution (0.3M) to encourage the presence of reactive unbridged thiol groups. After LCMS confirmed complete reaction, water was removed via lyophilisation to give complex **Tb.L_0_** (104 mg, quant.) as a pale pink solid. **IR (***v_max_***/cm^-1^, neat):** 1/λ 3393 (O-H stretch), 2987, 2939, 2873 (alkane C-H stretch), 1680, 1593 (**O=C**-OH and **O=C**-O-Tb stretch). **HPLC** [H2O (0.1% v/v HCOOH)/CH3OH(0.1% v/v HCOOH)]: **t_R_** = 6.656 minutes, 46.6% CH_3_OH. **LCMS:** m/z = 698 corresponding to the molecular ion [M+H^+^]. **HRMS-ESI:** [M+Na^+^]: Calculated = 720.1117 g/mol, seen = 720.1118 g/mol.

**Figure S6.** Analytical HPLC trace of **Tb.L_0_** moving from 100% acidified water to 100% acidified methanol over 14 minutes.

### **Complex Gd.L_0_**: Gadolinium(III) (1,4,7,10-Tetraazacyclododecane-1-(methyl-m-3-[mercaptopropionic acid])-4,7,10-triacetic acid)

Complex **Gd.NO_2_** (~60 mg, estimated 0.06 mmol, 1 equiv.) was dissolved in HEPES solution (5 mM, 8 mL). 3-Mercaptopropionic acid (9 µL, 0.10 mmol, 2 equiv.) was added and the solution was stirred at 37^°^C for 18 h. The pH was regularly adjusted to pH 7 with the use of sodium hydroxide solution (0.3M) to encourage the presence of reactive unbridged thiol groups. After LCMS confirmed complete reaction, water was removed via lyophilisation to give the crude complex **Gd.L_0_** (121 mg, quant.) as a pale cream solid. **IR (***v_max_***/cm^-1^, neat):** 3357, 2931, 1664, 1586, 1389, 1201, 1119, 1084, 799, 719, 663, 499. **HPLC** [H2O (0.1% v/v HCOOH)/CH_3_OH(0.1% v/v HCOOH)]: t_R_ = 5.753 minutes, 37.5% CH_3_OH. **HRMS-ESI:** [M+Na^+^]: Calculated = 719.1105 g/mol, seen = 719.1105 g/mol.

**Figure S7.** Analytical HPLC trace of **Gd.L_0_** moving from 100% acidified water to 100% acidified methanol over 10 minutes.

## 3. Synthesis of Macromolecular Imaging Agents

### Synthesis of block copolymer particles containing Ln.L

Here, the synthesis of particles containing Gd.L in the particle shell and Tb.L_2_ in the particle core: Shell-Gd.L_2_+Core-Tb.L, is described. Particles containing only one complex or both complexes in the particle shell used an almost identical method. The DPs shown in the figures are target degrees of polymerisation, however, often NMR analysis indicated slightly longer degrees of polymerisation which were considered in the following steps. The NMR spectra are similar to that of the copolymers without Ln(III) however with some broadening seen.

### 3.1 Copolymerisation of OEGMEM and **Gd.L** to generate **PEG-Gd.L** precursor

To a stirred 80:20 water/DMSO mixture (3.275 mL) in a 10 mL flask was added OEGMEM500 (1050 µL, 2.27 mmol, 45 equiv.), trioxane (34 mg, 0.38 mmol), CTP (13.95 mg, 0.05 mmol, 1 equiv.), AIBA (273 uL of 10 mg/mL stock solution, 0.01 mmol, 0.2 equiv.) and Gd.L_2_ (8.6 mg, 0.012 mmol, 0.2 equiv.). The solution was purged with nitrogen for 5 minutes, before being stirred at 70 ^°^C for 3 hours (96% monomer conversion). The product was purified by dialysis (6 x 4 h, 3500 MWCO, 4 L deionised water) and lyophilized to yield a viscous pink oil. **^1^H NMR (400 MHz, D2O) (ppm):** 8.0-7.6 (5H, Aromatic end group), 4.2 (96H, COOC**H**_2_ side chains), 3.7 (1990HO-C**H**_2_C**H**_2_-O side chains), 3.4 (173H, OC**H**_3_ end groups), 1.9-0.9 (298H, p(OEGMEM) backbone **H**s). **NMR determined DP:** 56. **GPC data** (Agilent 1260 infinity, CHCl_3_/TEA 98:2 v/v, RID detector): M_n_ = 18910 g mol^-1^, M_w_ = 21395 g mol^-1^, *Ð* = 1.13. **DLS:** Z-Average = 6.9 nm, PDI = 0.1.

### 3.2 Chain Extension with OEGMEM to generate **PEG-Gd.L**

To a solution of p(OEGMEM)_56_-*st*-p(Gd.L_2_)_0.5_ (982 mg, 0.035 mmol, 1 equiv.) in an 80:20 water/DMSO mixture (2.35 mL) was added OEGMEM500 (60 µL, 0.17 mmol, 5 equiv.), trioxane (0.97 mg, 0.01 mmol), AIBA (135 µL of 10 mg/mL stock solution, 0.007 mmol, 0.2 equiv.). The solution was purged with nitrogen for 5 minutes, before being stirred at 70 ^°^C for 2.5 hours (56 % conversion by NMR) at which point the reaction mixture was very viscous. The product was purified by dialysis (6 x 4 h, 3500 MWCO, 4 L deionised water) and lyophilized to yield a viscous pink oil. **^1^H NMR (400 MHz, D2O) (ppm):** 8.0-7.9 (5H, Aromatic end group), 4.2 (99H, COOC**H**_2_ side chains), 3.7 (2130H, O-C**H**_2_C**H**_2_-O side chains), 3.6 (181H, OC**H**_3_ end groups), 1.8-0.9 (337H, p(OEGMEM) backbone **H**s). **NMR determined DP:** 60. **GPC data** (Agilent 1260 infinity, CHCl_3_/TEA 98:2 v/v, RID detector): M_n_ = 20712 g mol^-1^, M_w_ = 24481 g mol^-1^, *Ð* = 1.18. **DLS:** Z-Average = 7.3 nm, PDI = 0.1.

**3**.3 RAFT-Emulsion Polymerisation: Synthesis of **Shell-Gd.L+Core-Tb.**L

Prepare a 50 mg/mL stock solution of AIBA in water. In this exact order, [p(OEGMEM)_50_-*st*-p(Gd.L_1_)_0.2_]-*b*-p(OEGMEM)_5_ (992 mg, 0.024 mmol, 1 equiv.), trioxane (45 mg, 0.5 mmol), AIBA (26 µL of stock solution, 0.0048 mmol, 0.2 equiv.) were added to water (11.33 mL). The solution was adjusted to pH 6 using NaOH (2 M). Whilst stirring, MMA (638 µL, 6 mmol, 250 equiv.) was added dropwise. The solution was purged with nitrogen for 10 minutes, then heated at 70 ^°^C for 30 minutes when the solution turned cloudy, at which point EGDMA (10 µL, 0.054 mmol, 2.3 equiv.) and Tb.L_2_ (4 mg, 0.005 mmol, 0.2 equiv.) dissolved in 1 mL water was added. The solution was heated for a further 1.5 h, before purification by dialysis (6 x 4 h, 3500 MWCO, 4 L deionised water) to give the product, a cloudy pink solution. **IR (*v*_max_/cm^-1^, neat):** 2868, 1725, 1449, 1097, 1037, 948, 849. **DLS: Z-Average:** 30.40 nm, PDI: 0.081.

|  (a)  (b)  (c) | **Scheme S1**. The three possible routes to synthesize **Ln.L**-containing particles, showing the mCTA on the left being chain extended with MMA, EGDMA and in some cases **Ln.L** to generate (a) **Shell-Ln.L**, (b) **Core-Ln.L**, (c) **Shell-Ln.L+Core-Ln.L**. The crosslinker (green) and **Ln.L** were added 0.5 h into the reaction. |
| --- | --- |

## 4. Characterisation of Ln.L-Containing Macromolecules

### GPC Analysis of Ln.L-containing Macromolecules

| ***Table S3.*** *GPC data for* ***PEG-Ln.L*** *compounds.* | | |
| --- | --- | --- |
| Sample | M_w_ (g/mol) | *Đ* |
| **PEG-Gd.L** | 24481 | 1.18 |
| **PEG-Tb.L** | 27042 | 1.19 |
| **PEG-Gd.L+Tb.L** | 23800 | 1.18 |
| **PEG-Gd.L_1_** | 61720 | 1.85 |

|  |  |
| --- | --- |
| **Figure S8.** GPC traces of **PEG-Gd.L** (left) and **PEG-Gd.L**+**Tb.L** (right). | |
|  \| ; **Figure S9.** GPC traces of **PEG-Tb.L** (left) and **PEG-Gd.L_1_** (right). \| | |

### DLS Analysis of Ln.L-containing Macromolecules

| **Table S4.** A summary of DLS analysis of particles containing imaging agents, showing average diameter and PDI. | | | | |
| --- | --- | --- | --- | --- |
| **Particle Sample *^a^*** | **DLS** | | **TEM *^d^*** | **AFM *^f^*** |
|  | **d ^b^, (nm)** | **PDI *^c^*** | **(nm)** | **(nm)** |
| Shell-**Tb.L** | 59 | 0.02 | 45 ± 4 | 28 ± 4 |
| Core-**Tb.L** | 45 | 0.04 | 50 ± 3 | 27 ± 3 |
| Shell-**Gd.L** | 19 | 0.16 | ^e^ | ^g^ |
| Core-**Gd.L** | 22 | 0.16 | ^e^ | ^g^ |
| Shell-**Tb.L**+**Gd.L** | 23 | 0.12 | ^e^ | 14 ± 2 |
| Core-**Tb.L**+Shell-**Gd.L** | 30 | 0.08 | 25 ± 2 | 18 ± 3 |
| Shell-**Gd.L_1_** | 115 | 0.01 | 101 ± 4 | 69 ± 9 |
| Core-**Gd.L_1_** | 35 | 0.07 | ^e^ | 16 ± 3 |
| ^a^ Particle names in note form correspond to the schematics described in Figure 4.1. ^b^ Z average diameter by DLS. ^c^ Average PDI of the DLS measurements over three runs. ^d^ The average diameter of 25 particles (where possible) was measured using TEM images, and the standard deviation related to this value. ^e^ = not possible to measure particles in TEM image due to low image quality or particle not visible. ^f^ The average diameter of 50 particles was measured using AFM images, and the standard deviation related to this value. ^g^ Particles not visible / not spherical. | | | | |

**Figure S10**. **Shell-Tb.L**: DLS spectrum (left), DLS correlogram (right).

**Figure S11.** **Core-Tb.L**: DLS spectrum (left), DLS correlogram (right).

**Figure S12**. **PEG-Tb.L**: DLS spectrum (left), DLS correlogram (right).

|  |  |
| --- | --- |
| **Figure S13**. **Shell-Gd.L**: DLS spectrum (left), DLS correlogram (right). | |
|  |  |
| **Figure S14.** **Core-Gd.L**: DLS spectrum (left), DLS correlogram (right). | |

|  |  |
| --- | --- |
| **Figure S15.** **Shell-Gd.L_1_**: DLS spectrum (left), DLS correlogram (right). | |
|  |  |
| **Figure S16.** **Core-Gd.L_1_**: DLS spectrum (left), DLS correlogram (right). | |
|  | |
| **Figure S17.** **Shell-Gd.L**+**Tb.L**: DLS spectrum (left), DLS correlogram (right). | |
|  | |
| **Figure S18.** **Shell-Gd.L**+Core-**Tb.L**: DLS spectrum (left), DLS correlogram (right). | |
|  | |

**Figure S19**. AFM images of macromolecules containing **Ln.L.**

|  |  |
| --- | --- |
| (a). **Shell-Gd.L**, particles do not appear spherical. | (b). **Core-Gd.L**, no visible particles. |
|  |  |
| (c). **Shell-Tb.L**, particles appear to coalesce. | (d). **Core-Tb.L**, spherical particles. |
|  |  |
| (e). **Shell-Gd.L+Tb.L**, particles appear to coalesce. | (f). **Shell-Gd.L+Core-Tb.L**, spherical particles. |
|  |  |
| (g). **Shell-Gd.L_1_**, spherical particles. | (h). **Core-Gd.L_1_**, particles appear to coalesce. |
|  | |

### ICP-MS Analysis

| ***Table S5.*** *A summary of the ICP-MS data generated* ***Tb.L*** *and dual imaging particles and determination of the concentration of the original undiluted particle sample.* | | | | | |
| --- | --- | --- | --- | --- | --- |
| Sample | **PEG-Tb.L** | **Shell-Tb.L** | **Core-Tb.L** | **Shell-Tb.L+Gd.L** | **Core-Tb.L+Shell-Gd.L** |
| Tb(III) Signal / cps *^a^* | 2253585.07 | 4753373.9 | 2993489.1 | 3579603.2 | 3540032.0 |
| Tb(III) ICP-MS Sample / ppb *^b^* | 2.30 | 5.07 | 3.12 | 3.8 | 3.7 |
| Tb(III) Undiluted sample / ppb *^c^* | 8053.26 | 17739.54 | 10920.29 | 13191.4 | 13038.1 |
| Tb Undiluted sample/ mM *^d^* | 0.050 | 0.11 | 0.069 | 0.083 | 0.082 |
| Gd(III) Signal / cps *^a^* |  |  |  | 1346138.7 | 1135427.2 |
| Gd(III) ICP-MS Sample / ppb *^b^* |  |  |  | 8.14 | 6.85 |
| Gd(III) Undiluted sample / ppb *^c^* |  |  |  | 28486.7 | 23976.0 |
| Gd(III) Undiluted sample/ mM *^d^* |  |  |  | 0.18 | 0.15 |
| *^a^ The cps signal generated from the ICP-MS measurement. ^b^ Input of the cps signal into the calibration equation allowed calculation of the ICP-MS sample concentration. ^c^ The ICP-MS sample concentration was much more dilute than the original particle solution, so this is corrected for here by multiplying the ICP-MS sample concentration by its dilution factor: 3500. ^d^ The concentration of the undiluted particle sample is converted from units of ppb to mM.* | | | | | |

| ***Table S6.*** *A summary of the Tb(III) and Gd(III) concentrations determined by ICP-MS compared to the theoretical concentrations of the lanthanides if 100% incorporation was achieved.* | | | | | | |
| --- | --- | --- | --- | --- | --- | --- |
| **Particle Sample** | **[Tb(III)]_theo_**  **(mM)** | **[Tb(III)]_actual_**  **(mM)** | **% Tb(III) loading** | **[Gd(III)]_theo_**  **(mM)** | **[Gd(III)]_actual_**  **(mM)** | **% Gd(III) loading** |
| Shell-**Tb.L** | 0.22 | 0.11 | **50** | - | - | - |
| Core-**Tb.L** | 0.22 | 0.069 | **31** | - | - | - |
| Shell-**Tb.L**+**Gd.L** | 0.14 | 0.083 | **59** | 0.39 | 0.18 | **46** |
| Core-**Tb.L**+Shell-**Gd.L** | 0.18 | 0.082 | **46** | 0.31 | 0.15 | **48** |
|  | | | | | | |
|  | | | | | | |

### UV-Vis Analysis of Tb.L and Tb.L-containing Macromolecules

|  | |
| --- | --- |
| **Figure S20.** UV-Vis absorbance spectra for polymer systems containing **Tb.L**, measured in water (pH 7.0, 295K). UV-Vis absorbance spectra were conducted at 10 × dilution of the purified particle solution. | |
|  |  |
| **Figure S21.** UV-Vis and emission spectra of **Tb.L** (left), and extinction coefficient determination (right). Conducted in water at pH 7.0, 295 K. Emission spectra used sample at 0.01 absorbance, λ_exc_ = 278 nm. | |
|  | |

|  |  |
| --- | --- |
|  |  |
| **Figure S22.** UV-Vis absorbance spectra of (a) **PEG-Gd.L+Tb.L,** (b) **Shell-Gd.L**+**Tb.L,** (c) **Shell-Gd.L+Core-Tb.L**, and (d) p(OEGMEM)_25_ and the chain transfer agent for comparison. Recorded in water at pH 7.0, 295 K, using a 10-fold dilution of the synthesised polymer sample. | |

### Emission Spectra of Ln.L-containing Macromolecules

|  |  |
| --- | --- |
| **Figure S23.** (left) Steady-state emission spectra of **Shell-Tb.L** (λ_exc_ = 282 nm) at particle synthesis concentration, and (right) time-resolved emission spectra of **Shell-Tb.L** at 100x dilution, each measured in water at pH. 7.0, 295 K. | |
|  |  |
| **Figure S24**. (left) Steady-state emission spectra of **CoreTb.L** (λ_exc_ = 282 nm) at particle synthesis concentration, and (right) time-resolved emission spectra of **Core-Tb.L** at 100x dilution, each measured in water at pH. 7.0, 295 K. | |
|  |  |
| **Figure S25.** (left) Steady-state emission spectra of **PEG-Tb.L** (λ_exc_ = 282 nm) at particle synthesis concentration, and (right) time-resolved emission spectra of **PEG-Tb.L** at 100x dilution, each measured in water at pH. 7.0, 295 K. | |

### Emission Decay Curves of Tb.L-containing Macromolecules in H_2_O and D_2_O

|  |  |
| --- | --- |
| **Figure S26.** Luminescence decay curves of **Shell-Tb.L** in H_2_O (left), D_2_O (right), measured at 295 K. | |
|  |  |
| **Figure S27.** Luminescence decay curves of **Core-Tb.L** in H_2_O (left), D_2_O (right), measured at 295 K. | |
|  |  |
| **Figure S28.** Luminescence decay curves of **PEG-Tb.L** in H_2_O (left), D_2_O (right), measured at 295 K. | |

### Relaxivity Analysis

The ^1^H Nuclear Magnetic Relaxation Dispersion (NMRD) profiles were carried out at three different temperatures (283, 298 and 310 K) on a Fast-Field Cycling (FFC) Stelar SmarTracer Relaxometer over a range of magnetic field strengths from 0.00024 to 0.25 T (corresponding to 0.01–10 MHz proton Larmor Frequencies). The relaxometer operates under computer control with an absolute uncertainty in 1/*T*_1_ of ±1%. Additional data points in the range 20–120 MHz were obtained on a High Field Relaxometer (Stelar) equipped with the HTS-110 3T Metrology Cryogen-free Superconducting Magnet. The measurements were performed using the standard inversion recovery sequence (20 experiments, 4 scans) with a typical radio frequency 90° pulse length of 3.5 μs. The reproducibility of the data was within ±0.5%. The temperature was controlled with a Stelar VTC-91 heater airflow equipped with a copper-constantan thermocouple (uncertainty of ±0.1 K). The diamagnetic contribution was measured by collecting ^1^H NMRD profiles of the unloaded nanoparticles, at different temperatures. The relaxivity (*r*_i_, mM^−1^ s^−1^) at different magnetic fields and temperatures was obtained by measuring the relaxation rates of the paramagnetic systems, subtracting the diamagnetic contribution of the polymeric nanoparticle, and normalizing the final value to mM concentration of Gd(III).

^17^O-NMR *T*_2_ data were carried out on a Bruker Avance III Spectrometer equipped with a wide bore 11.7 Tesla magnet. The spectrometer is equipped with a 5 mm double resonance Z-gradient broadband probe and with a Bruker BVT-3000 unit for temperature control. Aqueous solutions of the samples at physiological pH (7.4) and containing 2.0% of the ^17^O isotope (Cambridge Isotope) were used.

*Discrete Gd(III) complexes*

|  |  |
| --- | --- |
| **Figure S29.** NMRD profile of **Gd.L** (left) and **Gd.L_0_** (right). **Gd.L_1_** was previously synthesised and reported by Berki *et al*.^[5]^ | |
|  |  |
| **Figure S30.** ^17^O NMR (500 MHz) profiles for the **Gd.L_0_** complex (15 mM in water at pH 7.4). | |

*Gd(III) Complexes Incorporated into Macromolecules*

|  |  |
| --- | --- |
| **Figure S31.** NMRD of PEG-**Gd.L_1_** (left) and Shell-**Gd.L_1_** (right). | |
|  | |
| **Figure S32**. NMRD profile of **Core-Gd.L_1_**, lacking a peak in the expected 10-100 MHz region. | |

**Figure S33.** Variable temperature experiments on **Gd.L**-containing macromolecules, where: Blue: **Shell-Gd.L**; Red: **PEG-Gd.L**

*Dual Imaging Macromolecules*

|  **(a) Shell-Gd.L+Tb.L** |  **(b) Shell-Gd.L+Tb.L** |
| --- | --- |
|  **(c) PEG-Gd.L+Tb.L** |  **(d) Shell-Gd.L+Core-Tb.L**  |
| **Figure S34.** NMRD profiles of polymers and particles containing both **Gd.L_­_** and **Tb.L**. | |

| **Table S7.** Gd(III) concentration in macromolecules. | |
| --- | --- |
| Sample | ICP-MS Concentration (mM) |
| Core-**Gd.L_1_** | 0.07 |
| Shell-**Gd.L_1_** | 0.72 |
| Core-**Gd.L** | 0.32 |
| *ICP-MS measurements of Gd(III)-containing macromolecules prior to relaxivity analysis.* | |

| **Table S8.** T1 relaxation times of Tb(III)-containing macromolecules compared to water. | |
| --- | --- |
| Sample | T1 Relaxation time / ms |
| Shell-**Tb.L** | 1600 |
| Core-**Tb.L** | 1500 |
| PEG-**Tb.L** | 1200 |
| Shell-**Gd.L**+**Tb.L** | 341 |
| Shell-**Gd.L**+Core-**Tb.L** | 439 |
| Water | 900 |
| *Analysis conducted as preliminary results on a Bruker MiniSpec at Loughborough university. Concentrations were used as determined by ICP-MS. 200μL sample volumes, 37°C. Note: some issues with the equipment prevented calibration so these results are only speculative, and this test was designed to determine whether the Tb(III) containing samples appeared to express any increase in relaxation rate compared to pure water.* | |

*Dual Imaging Macromolecules Photophysical Analysis*

|  |  |
| --- | --- |
| **Figure S35.** Time-resolved emission spectra of dual imaging particles: (left) **Shell-Gd.L**+**Tb.L,** and (right) **Shell-Gd.L+Core-Tb.L,** measured in water at pH 7.0 and 295 K, excited at 278 nm. | |

**Shell-Gd.L+Tb.L	Shell-Gd.L+Core-Tb.L**

**Figure S36.** Comparison of emission intensity (at 546 nm) for the **Tb.L-**containing particles after 10-fold dilution, measured in water at pH 7.0 and 295 K. Emission values are normalised to the ICP-MS-calculated Tb(III) concentrations.

|  |  |
| --- | --- |
| **Figure S37.** The minimal influence of pH on the diameter and emission of dual imaging particles: (left) **Shell-Gd.L+Tb.L,** and (right) **Core-Tb.L+Shell-Gd.L**_._ | |

| **Table S9.** A summary of the relaxivity values of (a) discrete Gd(III) complexes, (b) linear NAM-based polymers, (c) and (d) block copolymer particles and (e) dual imaging systems with comparison to their molecular weights and diameters as measured by GPC and DLS. | | | | |
| --- | --- | --- | --- | --- |
| Sample | r_1_ | | M_w_  (g/mol) | Diameter (nm) |
|  | 298 K | 310 K |  |  |
| Gd.L_0_ | 5.1 | 4.2 | 696 | -- |
| Gd.L | 5.7 | 4.8 | 736 | -- |
| Gd.L_1_ | 6.5 | 5.1 | 638 | -- |
| PEG-Gd.L | 18.2 | 19.3 | 24481 | 7.27 |
| Shell-Gd.L | 23.7 | 25.5 | -- | 19.26 |
| Core-Gd.L | 18.0 | 20.5 | -- | 21.95 |
| PEG-Gd.L_1_ | 24.1 | 21.0 | 61720 | -- |
| Shell-Gd.L_1_ | 25.8 | 26.7 | -- | 115.1 |
| Core-Gd.L_1_ | 17.6 | -- | -- | 34.94 |
| PEG-Gd.L+Tb.L | 16.0 | 17.0 | 23800 | 6.45 |
| Shell-Gd.L+Tb.L | 30.2 | 33.0 | -- | 22.54 |
| Shell-Gd.L+Core-Tb.L | 15 | -- | -- | 30.4 |
| *Relaxivity values given for 32 MHz. M_w_ measured by GPC; this may be unrepresentative for some samples with high dispersities.* | | | | |

### Appendix

**Figure S38.** ^1^H NMR spectrum of 2-hydroxymethyl-4-nitropyridine in DMSO-*d_6_*, solvent peak at 2.50 ppm.

**Figure S39.** ^13^C NMR spectrum of 2-hydroxymethyl-4-nitropyridine in DMSO-*d_6_*, solvent peak at 39.52 ppm.

**Figure S40.** IR spectrum of 2-hydroxymethyl-4-nitropyridine.

**Figure S41.** ^1^H NMR Spectrum of DO3A in CDCl_3,_ solvent peak at 7.26 ppm.

**Figure S42.** ^13^C NMR Spectrum of DO3A in CDCl_3,_ solvent peak at 77.16 ppm.

**Figure S43.** IR Spectrum of DO3A.

**Figure S44.** ^1^H NMR Spectrum of crude Methyl-m-3-nitropyridine sulfonate methyl ester in CDCl_3,_ solvent peak at 7.26 ppm. This product is highly reactive and so was quickly used in the next step.

**Figure S45.** ^13^C NMR Spectrum of crudeMethyl-m-3-nitropyridine sulfonate methyl ester in CDCl_3,_ solvent peak at 77.16 ppm . This product is highly reactive and so was quickly reacted in the next step.

**Figure S46.** ^1^H NMR Spectrum of 1,4,7,10-Tetraazacyclododecane-1-(methyl-m-3-nitropyridine)-4,7,10-tris(t-butyl acetate) in CDCl_3,_ solvent peak at 7.26 ppm.

**Figure S47.** ^13^C NMR Spectrum of 1,4,7,10-Tetraazacyclododecane-1-(methyl-m-3-nitropyridine)-4,7,10-tris(t-butyl acetate) in CDCl_3,_ solvent peak at 77.16 ppm.

**Figure S48.** IR Spectrum of 1,4,7,10-Tetraazacyclododecane-1-(methyl-m-3-nitropyridine)-4,7,10-tris(t-butyl acetate).

**Figure S49.** ^1^H NMR Spectrum of 1,4,7,10-Tetraazacyclododecane-1-(methyl-m-3-nitropyridine)-4,7,10-triacetic acid in D_2_O, solvent peak at 4.80 ppm.

**Figure S50.** IR Spectrum of 1,4,7,10-Tetraazacyclododecane-1-(methyl-m-3-nitropyridine)-4,7,10-triacetic acid.

**Figure S51.** IR Spectrum of **Gd.NO_2_**.

**Figure S52.** High-resolution mass spectrometry (ESI) of **Gd.NO₂** showing an observed [M+H]⁺ at m/z 660.1023, in agreement with the theoretical mass of 660.1024. The experimental isotopic distribution is consistent with the calculated pattern. m/z values of small impurities and generated chemical formulas are also shown.

**Figure S53.** IR Spectrum of **Tb.NO_2_**.

**Figure S54.** IR Spectrum of **Gd.L.**

**Figure S55.** IR Spectrum of **Tb.L**.

**Figure S56.** Comparison of ¹H NMR spectra of **Tb.NO₂** (green) and **Tb.L** (blue), recorded in D₂O at 500 MHz, showing the spectral changes upon conversion.

### Representative ^1^H-NMR Spectra of Particles

**Figure S57.** ^1^H NMR spectrum of **Shell-Tb.L+Gd.L** after two hours of reaction, demonstrating minimal MMA monomer present at c.a. 5.5 and 6.1 ppm.

**Figure S58.** ^1^H NMR spectrum of **Shell-Gd.L+Core-Tb.L** after two hours of reaction, demonstrating minimal MMA monomer present at c.a. 5.5 and 6.1 ppm.

**Figure S59.** ^1^H NMR of **PEG-Tb.L**: [p(OEGMEM)_45_-st-p(Tb.L)_0.1_]-b-p(OEGMEM)_5,_ where DPs given are target DPs.

**Figure S60.** ^1^H NMR of **PEG-Gd.L**: [p(OEGMEM)_45_-st-p(Gd.L)_0.1_]-b-p(OEGMEM)_5_, where DPs given are target DPs.

### **References**

[1]	C. J. Marsden, C. Breen, J. D. Tinkler, T. R. Berki, D. W. Lester, J. Martinelli, L. Tei, S. J. Butler and H. Willcock, “Crosslinked p(MMA) particles by RAFT emulsion polymerisation: tuning size and stability”, *Polym. Chem.*, **2022**, *13*, 4124-4135.

[2]	K. Suzuki, A. Kobayashi, S. Kaneko, K. Takehira, T. Yoshihara, H. Ishida, Y. Shiina, S. Oishi and S. Tobita, “Reevaluation of absolute luminescence quantum yields of standard solutions using a spectrometer with an integrating sphere and a back-thinned CCD detector”, *Phys. Chem. Chem. Phys.*, **2009**, *11*, 9850-9860.

[3]	A. Beeby, I. M. Clarkson, R. S. Dickins, S. Faulkner, D. Parker, L. Royle, A. S. de Sousa, J. A. Gareth Williams and M. Woods, “Non-radiative deactivation of the excited states of europium, terbium and ytterbium complexes by proximate energy-matched OH, NH and CH oscillators: an improved luminescence method for establishing solution hydration states”, *J. Chem. Soc., Perkin Trans. 2*, **1999**, *0*, 493-504.

[4]	K. L. Gempf, S. J. Butler, A. M. Funk and D. Parker, “Direct and selective tagging of cysteine residues in peptides and proteins with 4-nitropyridyl lanthanide complexes”, *Chem. Commun.*, **2013**, *49*, 9104-9106.

[5]	T. R. Berki, J. Martinelli, L. Tei, H. Willcock and S. J. Butler, “Polymerizable Gd(iii) building blocks for the synthesis of high relaxivity macromolecular MRI contrast agents”, *Chem. Sci.*, **2021**, *12*, 3999-4013.
